# Structurally Complex Precipitates Enhance Strength‐Ductility Synergy in a Duplex Medium Entropy Alloy

**DOI:** 10.1002/advs.75787

**Published:** 2026-05-22

**Authors:** Shaohua Gao, Yang Yang, Xiaoxuan Fan, Jinyu Zhang, Shuaiyang Liu, Jiao Li, Hui Wang, Wenli Song, Gang Liu, Jun Sun

**Affiliations:** ^1^ State Key Laboratory For Mechanical Behavior of Materials Xi'an Jiaotong University Xi'an P. R. China; ^2^ College of Materials Science and Engineering Sichuan University Chengdu P. R. China; ^3^ Institute of High Energy Physics Chinese Academy of Sciences (CAS) Beijing P. R. China; ^4^ Spallation Neutron Source Science Center Dongguan P. R. China

**Keywords:** core–shell microstructures, deformation mechanisms, medium entropy alloys, multi‐component intermetallic precipitates, strength‐ductility synergy

## Abstract

Chemically homogeneous intermetallic nanoprecipitates (INPs), despite their high strength, are intrinsically hard and brittle and often suffer from glide‐plane softening, which can strengthen alloys but severely degrade uniform elongation. This strength–ductility trade‐off is further exacerbated at cryogenic temperatures by the inherent brittleness of bcc‐based phases in precipitate‐strengthened fcc/bcc duplex alloys. Here we propose a strategy that overcomes these limitations through the design of structurally complex INPs, i.e., the ductile B2 multicomponent INPs (MINPs) assembled with dispersive nanocores and a chemical‐heterogeneity shell, in duplex fcc/bcc Fe_58_Ni_16_Cr_16_Al_10_ (at%) medium‐entropy alloys (Fe‐MEAs). Within the bcc constituent, these coherent core–shell B2 MINPs serve the dual role of dislocation sources and obstacles, analogous to the ordinary incoherent B2 MINPs that trigger twinning‐induced plasticity in the fcc constituent, yet they are substantially more effective in load transfer for high yield strength and in self‐hardening for large uniform elongation. Critically, the core–shell nanostructure suppresses the glide‐plane softening typical of conventional INPs and promotes the activation of unusual ⟨111⟩ dislocation multiplication and interactions under cryogenic conditions. This work demonstrates a structural complexification strategy for designing self‐hardening MINPs, opening a pathway to ductile, high‐strength materials for advanced cryogenic structural applications.

## Graphical abstract

Strong‐yet‐ductile precipitation‐hardening alloys require novel microstructural complexity of precipitates in particularly at cryogenic condition, to constantly impart strain hardening of hard‐and‐brittle precipitates. The present work creates innovative structurally complex precipitates assembled with dispersed nanocores and a chemical‐heterogeneity shell. Multiple slip activates unusual dislocation multiplication and interactions that effectively suppress slip softening, thereby yielding superior cryogenic mechanical properties in a medium‐entropy alloy.

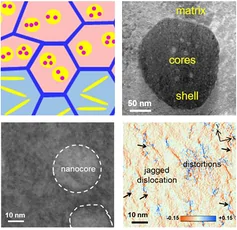

## 1 Introduction

Nanoprecipitation engineering is one of the most important strategy to improve the mechanical properties of alloys, because bifunctional nanoprecipitates can act as dislocation obstacles to strengthen alloys on the one hand and as dislocation sources to ductilize them on the other hand [1, 2]. The pursuit of high yield strength (YS, *σ*_y_) and large ductility (*ε*_f_) alloys reinforced by ordered intermetallic nanoprecipitates (INPs) has long been desirable via tuning the INP characters (e.g., shape, sizes, spacing, and so on) and synergistic deformation mechanisms of alloys to balance the conflict between strength and ductility, in particular at low temperatures [2, 3, 4, 5, 6]. Compared with the non‐deformable INPs associated with high strain hardening rates (SHRs, Θ) in the matrix [7], these deformable INPs can alleviate local stress concentrations to avoid microcracking for large ductility [8, 9] but would be sheared by gliding dislocations that transmit across the INP/matrix interface, leading to the glide plane softening phenomenon [10, 11]. In glide plane softening, the shearing of resistive precipitates by leading dislocations enables the following dislocations to easily glide on the same plane for progressive strain localization and thus reduced Θ. To enhance the strengthening effect of shearable INPs in alloys, a feasible way is to utilize precipitates with high antiphase boundary (APB) energies (γ_APB_), such as the ordered body‐centered‐cubic (bcc) B2‐NiAl in lieu of ordered face‐centered‐cubic (fcc) L1_2_‐Ni_3_Al INPs. In this regard, these deformable NiAl INPs (∼0.5 J/m^2^), with the 2.5‐fold higher γ_APB_ compared to Ni_3_Al (∼0.2 J/m^2^) [12, 13], emerge as superior candidates for pronounced ordering strengthening. For instance, Jiang et al. [12] designed the ultra‐strong Fe‐18Ni‐3Al‐4Mo‐0.8Nb‐0.08C‐0.01B (wt.%) maraging steels containing densely, fully coherent B2 INPs (∼2.7 ± 0.2 nm) to achieve the YS 1947 ± 23 MPa, uniform elongation (UE, 𝜀_u_) of ∼3.8%, and ductility 8.2% ± 0.7%.

Although traditionally ordered B2 INPs have high strength, their ductility is quite limited, especially at low temperatures, due to the lack of sufficient slip systems and inadequate SHRs Θ [14, 15]. Thus, numerous studies have been conducted to design strong‐yet‐ductile INPs via the non‐stoichiometric composition design [8, 13, 16] or C/B micro‐alloying [17, 18], in particular the high‐entropy design enables the multicomponent INPs (MINPs) overcome the inherent brittleness of traditional INPs [8, 9, 13, 15]. For example, these deformable B2 MINPs not only enhance the YS of multi‐principal‐element CoNiTiZrHf [15], FeCoNiAlTa [8], and CrCoNiAlTa [9] medium/high‐entropy alloys (M/HEAs), but also store abundant dislocations for their high SHRs and thus large UE, even at cryogenic temperatures [2, 9]. Compared with the single fcc‐phase matrix, their (fcc/bcc) duplex siblings indeed exhibit more superior properties at room temperature [19, 20, 21]. However, the disordered bcc‐phase in the matrix manifests cryogenic brittleness due to limited dislocation nucleation and multiplication caused by inefficient dislocation sources [22, 23]. As such, it is critical to devise a unique structure to mitigate the cryogenic brittleness by tailoring the B2 characters to deliver the bcc‐phase (dominant) alloys with a combination of high YS and large UE from the ambient to low temperatures.

To mitigate the cryogenic brittleness of the bcc matrix and simultaneously avoid the glide plane softening of traditional INPs at high strength levels, here we adopt the deformable and the non‐deformable B2 MINPs, that are respectively coherent with the bcc phase and incoherent with the fcc phase in a model duplex Fe_58_Ni_16_Cr_16_Al_10_ (at%) MEA (Fe‐rich MEA). The coherent B2 MINPs have a self‐organized structurally complex core–shell nanostructure (chemical‐heterogeneities B2 shell and disordered bcc nanocores), analogous to those observed in traditional Al–Mg–Sc [24] and Al–Sc–Zr [25] alloys, which can be attributed to the profuse fast‐diffusive Cr solutes in the bcc matrix but constrained in the fcc matrix (Refer to Note‐S1 and Figures S1 and S2 for more details). The bifunctional effects of coherent B2 MINPs, in terms of obstructing dislocation motion to strengthen the bcc matrix, and triggering dislocation multiplications to enhance their self‐hardening, is amplified by the structurally complex core‐shell nanostructure. Although the required critical resolved shear stress (CRSS) of <111> dislocations much higher than <100> dislocations in traditional B2 INPs [26, 27], abundant <111> dislocations are activated in coherent B2 MINPs. The chemical heterogeneities in the B2 shell effectively maintain a rugged energy landscape to adjust the dislocation motion and interactions under sluggish mobility at the same CRSS levels [23, 28]. At the same time, the bifunctional incoherent B2 MINPs give rise to severe stress concentrations to trigger deformation twins in the fcc matrix for enhanced SHRs. Overall, the improvement of strength and ductility can be simultaneously achieved by these synergistic deformation mechanisms. Our work conveys a transformative strategy for reconstructing precipitate nanostructures to endow them unexpected deformation behavior of strong‐and‐ductile metallic materials, in particular these cryogenic alloys.

## 2 Results

### 2.1 Microstructures

**FIGURE 1:**
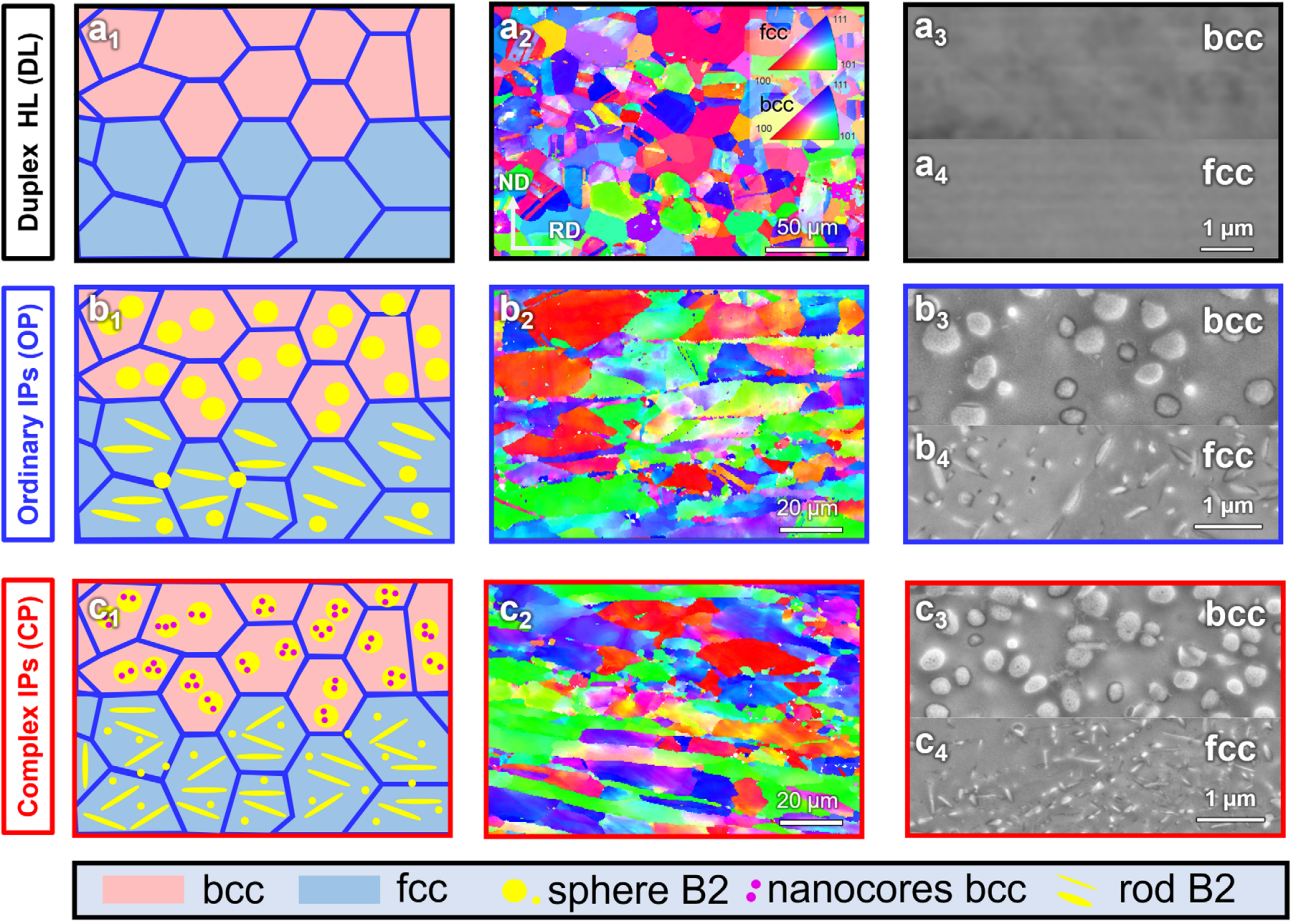
The characterization of three typical microstructures. (a_1_,b_1_,c_1_) Schematic maps of the microstructures of duplex heterogeneous micro‐lamellae structures (DL), ordinary MINPs (OP), and structurally complex MINPs (CP) samples. (a_2_,b_2_,c_2_) Corresponding EBSD inverse pole figure (IPF) maps of respective DL, OP, and CP samples. (a_3_,b_3_,c_3_) SEM images show the (no) MINPs in the bcc matrix of respective DL, OP, and CP samples. (a_4_,b_4_,c_4_) SEM images show the (no) MINPs in the fcc matrix of respective DL, OP, and CP samples.

The initial hot‐rolled Fe‐rich MEA with the composition of Fe_58_Ni_16_Cr_16_Al_10_ (at%), followed by water quenching (WQ), were annealed in 1200°C (30 min, followed by WQ), 900°C (1 h, followed by air cooling, AC), and 800°C (1 h, followed by AC), respectively, see Figure S3a. Three typical microstructures were introduced: the fcc/bcc duplex heterogeneous micro‐lamellae structure (DL, schematized as Figure 1a_1_), the ordinary B2 MINPs structure (OP, Figure 1b_1_), and the structurally complex B2 MINPs structures (CP, Figure 1c_1_). All the samples remain a laminated fcc/bcc duplex structure, verified from the phase distribution map (Figure S3b–d). There are higher fractions of the fcc phase ∼55%, ∼72%, and ∼60%, and average fcc grain sizes of ∼19.2, ∼15.1, and ∼11.8 µm in DL, OP, and CP alloys, respectively. The corresponding average bcc grain sizes are ∼29.7 µm in DL samples, ∼6.3 µm in OP samples, and ∼6.5 µm in CP samples (as shown in Figure 1a_2_,b_2_,c_2_). The phase boundaries between the fcc and bcc matrix mainly show the Kurdjumov–Sachs (K–S) orientation relationship (OR), i.e., {111}_fcc_//{110}_bcc_, <110>_fcc_//<111>_bcc_, indicated by the white line in Figure S3b–d.

**FIGURE 2:**
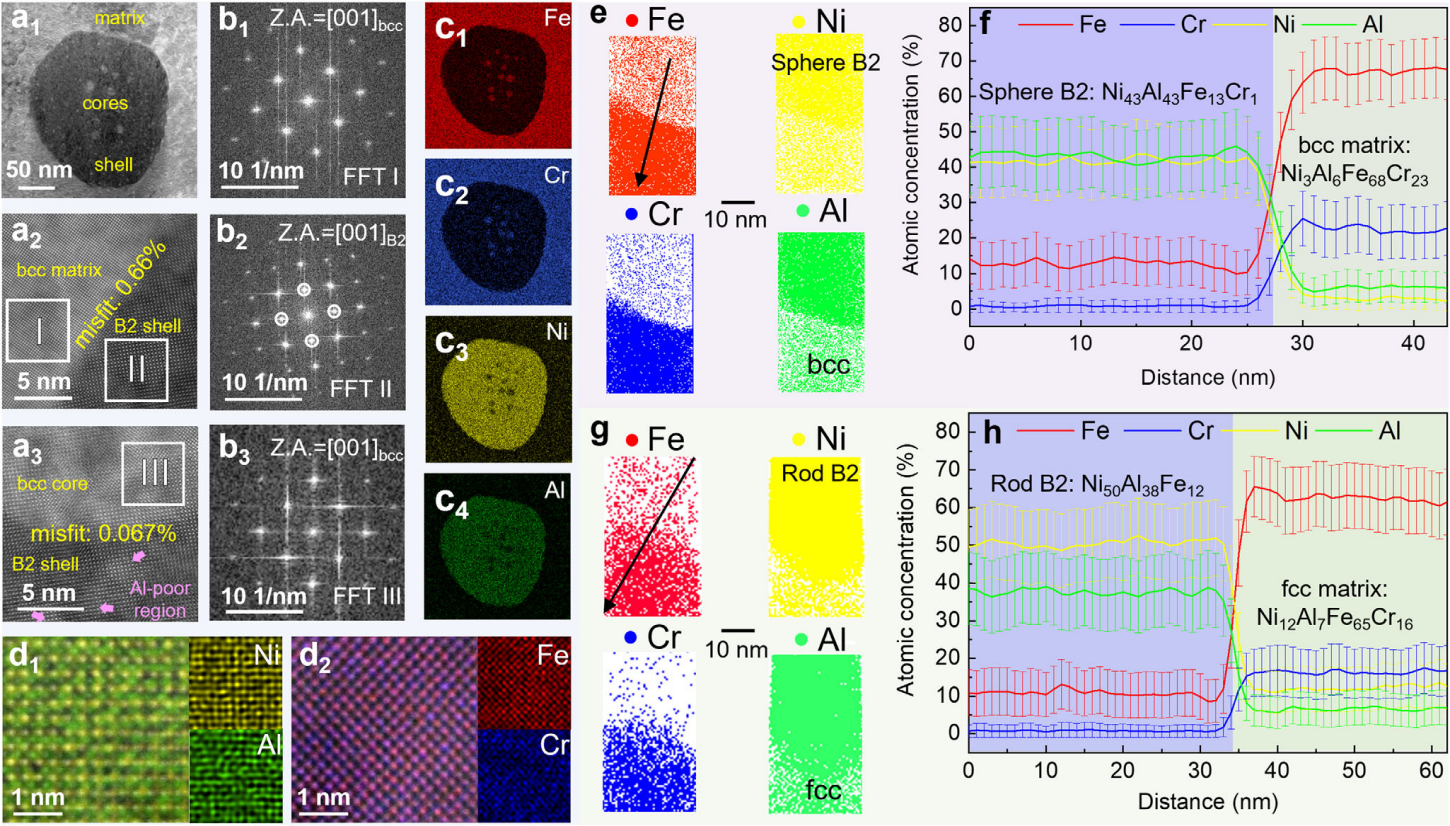
The microstructure of structurally complex MINPs in CP samples. (a_1_) An HAADF‐STEM image of a sphere precipitate in the matrix. (a_2_) An HAADF‐STEM image showing the interface between the bcc matrix and the shell. (a_3_) An HAADF‐STEM image showing the interface between the bcc core and the shell. (b_1_–b_3_) FFT images of regions I, II in (a_2_) and III in (a_3_), respectively. (c_1_–c_4_) EDS maps of sphere precipitates and the matrix. (d_1_) Atomic EDS maps of the shell. (d_2_) Atomic EDS maps of the nanocore. (e) The APT element distribution images in the bcc matrix. (f) One‐dimensional composition profiles across the bcc matrix and sphere B2 precipitates. (g) The APT element distribution images in the fcc matrix. (h) One‐dimensional composition profiles across the fcc matrix and rod B2 precipitates.

The associated inverse polar figure (IPF) maps reveal that the elongated bcc grains are preferentially oriented to the <101> direction along the rolling direction (RD), and fcc grains are randomly oriented, see Figure 1b_2_,c_2_. The kernel average misorientation (KAM) maps (see Figure S3e–g) show that the KAM value of the fcc matrix is slightly higher than that of the bcc matrix. Additionally, the spherical MINPs are introduced in the bcc matrix (see Figure 1b_3_,c_3_) and the rodded together with spherical MINPs are introduced in the fcc matrix (see Figure 1b_4_,c_4_), both in the OP and CP samples. By contrast, there are no MINPs in the DL sample (see Figure 1a_3_,a_4_). For the OP sample, in the bcc matrix, spherical precipitates have radius *R* = 129±9 nm, spacing *L* = 456±22 nm, volume fraction *V*_f_ = 14.4%. While in the fcc matrix, rod‐like precipitates have radius *R* = 87±14 nm, *L* = 427±37 nm, *V*_f_ = 10.3%, and aspect ratio (or the cross‐section length/width ratio) *c*/*a* = 4.87, and spherical precipitates have radius *R* = 43±14 nm, *V*_f_ = 2.2%. By contrast, in the CP sample, the sub‐micro spherical precipitates (*R* = 115±22 nm, *L* = 274±22 nm, *V*_f_ = 21.9%) contain densely dispersed nanocores (*R* = 15±22 nm, *L* = 45±22 nm, see Figure 2) in the bcc matrix. Meanwhile, densely small‐sized rod MINPs are precipitated with *R* = 34±5 nm, *L* = 183±22 nm, *V*_f_ = 14.6%, and *c*/*a* = 4.46, together with small spherical MINPs with *R* = 69±22 nm and *V*_f_ = 2.0% in the fcc matrix.

The formation of these dual‐morphology B2 precipitates is promoted by matrix recrystallization [7, 29]. In the non‐recrystallized structure, rod‐like B2 particles precipitate along shear bands [7]. The observation of the fcc and B2 interfaces also identifies the K‐S OR, see Figure S4. These interfaces promote anisotropic growth along {111} planes, yielding a rod‐like morphology [30]. In the recrystallized structure, grain boundaries with high stored energy act as fast diffusion channels for solute atoms, so B2 particles preferentially nucleate and grow at grain boundaries or triple junctions within the recrystallized grains [29]. These B2 particles are spherical or elliptical, appear as intragranular precipitates inside the recrystallized fcc grains, and possess relatively low interfacial energy.

Figure 2 shows structurally complex B2 MINPs in the CP alloy. These nanocores do not preferentially form at the matrix/precipitate interface but tend to form inside B2 to create the core‐shell nanostructure (Figure 2a_1_). Both coherent matrix/shell interface (lattice misfit: 0.66%, Figure 2a_2_) and the shell/core interface (lattice misfit: 0.067%, Figure 2a_3_) maintaining the cube‐on‐cube orientation relationship. The corresponding FFT patterns verify the matrix is bcc structured (Figure 2b_1_), the shell is B2 structured with the superlattice spots (Figure 2b_2_) and the nanocore is disordered bcc structured (Figure 2b_3_). The EDS maps (Figure 2c_1_–c_4_) further display the Fe/Cr‐rich bcc matrix, and the Ni/Al‐rich shell and Fe/Cr‐rich nanocores in a precipitate. There is one layer of Ni atoms by one layer of Al atoms alternately along the direction of [010] in the shell (Figure 2d_1_), which verifies its NiAl‐type of the ordered B2 structure. The chemical heterogeneities (Al‐poor, pink arrows in Figure 2a_3_) can be detected in the B2 shell and the elemental enrichment of nanocores is mainly Fe and Cr, showing a random structure where Fe is ∼60 at% and Cr is ∼40 at% (Figure 2d_2_). The exact compositions of the bcc matrix with sphere B2 (Figure 2e,f) and the fcc matrix with rod B2 (Figure 2g,h) were also determined by APT. The one‐dimensional concentration profiles (Figure 2f) demonstrate that the compositions of sphere precipitates (Ni_43_Al_43_Fe_13_Cr_1_: a typical NiAl‐type B2 phase where Ni: Al = 1:1) and the bcc matrix (Ni_3_Al_6_Fe_68_Cr_23_). The one‐dimensional concentration profiles across the fcc (Ni_12_Al_7_Fe_65_Cr_16_) matrix and the rod (Ni_48_Al_38_Fe_12_) precipitate also display their exact compositions (Figure 2h). The rod precipitates are incoherent with the fcc matrix with the lattice misfit of 3.20%, see Figure S4.

### 2.2 Mechanical Properties

**FIGURE 3:**
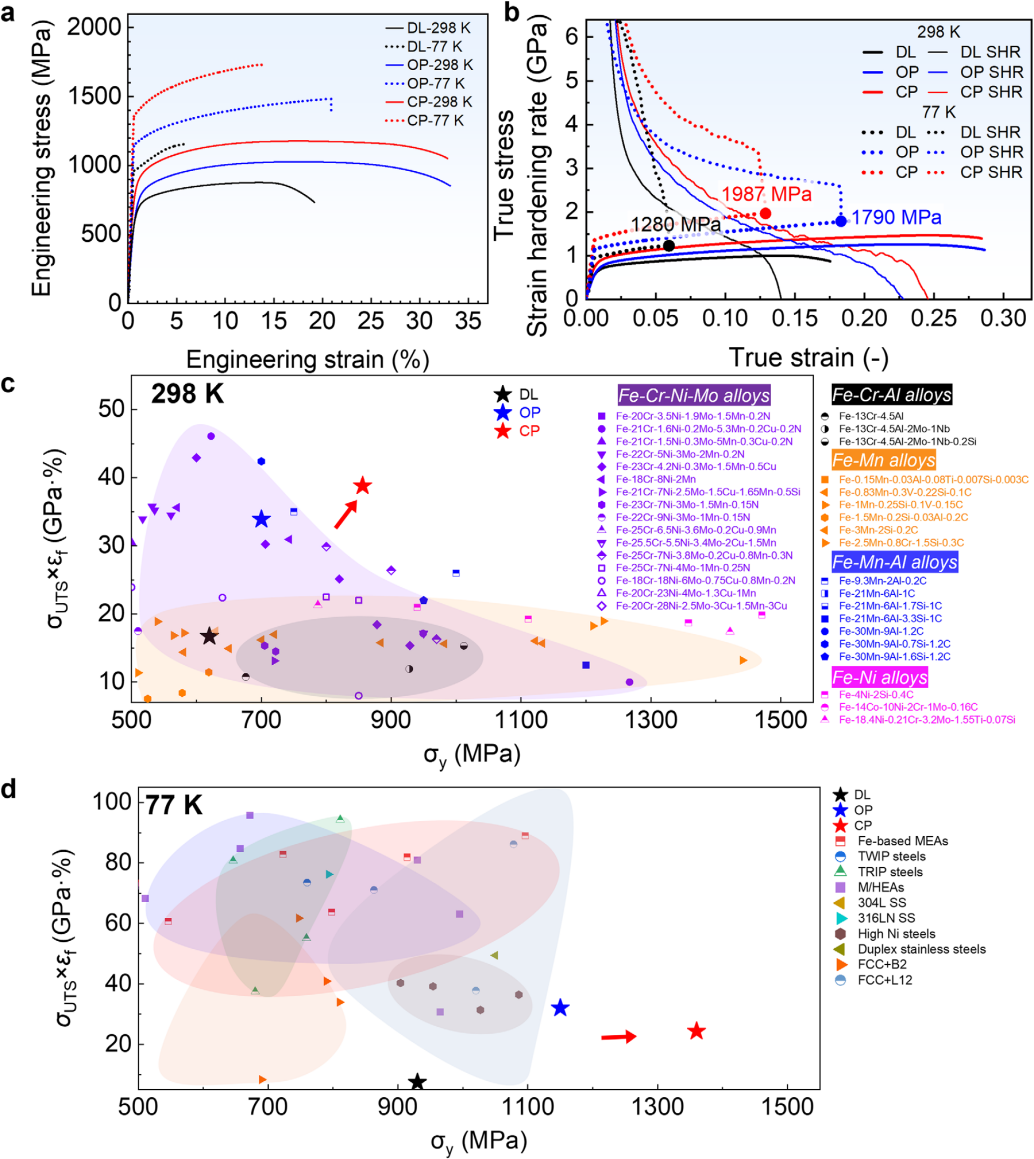
Mechanical properties. (a) Representative engineering strain‐stress curves of the DL, OP, and CP samples. (b) Corresponding true strain‐stress and strain hardening rate curves. (c) A comparison of *σ*_y_ vs. *σ*_UTS_×*ε*_f_ of the present alloys at 298 K with other advanced Fe‐rich alloys or steels, including Fe‐Ni (maraging steels) [31, 32, 33], Fe–Mn (TWIP/TRIP steels) [34, 35, 36, 37, 38], Fe–Cr–Al (precipitate‐strengthened ferrite steels) [39], Fe–Mn–Al (precipitate‐strengthened ultrahigh strength light steels) [40, 41, 42] and Fe–Cr–Ni–Mo (precipitate‐strengthened austenite or duplex stainless steels) [32, 43, 44, 45, 46, 47, 48, 49, 50, 51, 52, 53, 54, 55] alloys. See more details in Table S2. (d) A comparison of *σ*_y_ vs. *σ*_UTS_×*ε*_f_ of the present alloys at 77 K with other Fe‐based MEAs [56], TWIP steels [57], TRIP steels [58], 306L stainless steels [59], 316 L stainless steels [60], high Ni steels [61], duplex stainless steels [56, 62], and B2/L1_2_ precipitation strengthened H/MEAs [9]. See more details in Table S3.

Representative engineering stress‐strain curves of the DL, OP, and CP samples at 298 and 77 K are shown in Figure 3a, along with the true stress‐strain and their SHR curves in Figure 3b. At 298 K, the YS *σ*_y_ is 856±12 MPa (means ±1 standard deviation throughout this Article), together with an ultimate tensile strength (*σ*_UTS_) of 1176±15 MPa, a UE *ε*_u_ of 20%±1.2% and ductility *ε*_f_ of 33%±1.4% in the CP sample. All these values are the maximum among these three typical samples, where *σ*_y_ equals ∼620 and ∼700 MPa in the DL and OP samples, respectively. At 77 K, both *σ*_y_ and *σ*_UTS_ increase among these three samples. Unexpectedly, *σ*_y_ and *σ*_UTS_ increase to 1360±15 and 1740±20 MPa, respectively in the CP sample with a moderate compromising *ε*_u_ of 14%±1.0%. The true stress even reaches 1987 MPa at the true strain of 0.129 in the CP sample, attributed to the highest and stable SHR Θ (∼3.5 GPa) of the CP sample at 77 K. By contrast, *σ*_y_ increases to ∼930 and ∼1150 MPa in the DL and OP samples, respectively.

At 298 K, we observe a remarkable strength‐ductility combination, with *σ*_UTS_×*ε*_f_ reaching ∼40 GPa·% for the CP sample. These superior properties are compared in terms of *σ*_y_ vs. *σ*_UTS_×*ε*_f_ (Figure 3c) with Fe‐Ni maraging steels [31, 32, 33], Fe–Mn TWIP/TRIP steels [34, 35, 36, 37, 38], and precipitate‐strengthened steels, e.g., Fe–Cr–Al [39], Fe–Mn–Al [40, 41, 42] and Fe–Cr–Ni–Mo [32, 43, 44, 45, 46, 47, 48, 49, 50, 51, 52, 53, 54, 55]. The duplex Fe‐rich MEAs designed in this work exhibit an excellent balance of *σ*_y_ and *σ*_UTS_×*ε*_f_, surpassing the previous reported most precipitate‐strengthened alloys.

At 77 K, we compare the mechanical properties of our samples with some typical advanced cryogenic alloys in terms of *σ*_y_ vs. *σ*_UTS_×*ε*_f_ (Figure 3d), *ε*_u_ vs. *σ*_y_ (Figure S5a) and *ε*_u_ vs. *σ*_UTS_ (Figure S5b), including 304L/316LN steels [59, 60], high‐Ni steels [61], TWIP/TRIP steels [57, 58], duplex stainless steels [56, 62], B2/L1_2_ precipitation strengthened alloys [9], and some Fe‐MEAs and H/MEAs [56]. The present CP sample also shows an excellent cryogenic strength‐ductility combination. Unlike some of (duplex) TRIP steels/alloys subject to serrated plastic flow (*ε*_u_ is pseudo‐uniform) or yield drops at 77 K [56, 63], our alloys have stable plastic flows with truly UE *ε*_u_. Our Fe‐rich MEAs also show a comparable strength‐toughness trade‐off to most advanced existing (duplex) fcc/bcc alloys at 298 K, see more details in Note‐S2 and Figure S5c,d.

### 2.3 In Situ Tension Behavior and Deformed Substructures

**FIGURE 4:**
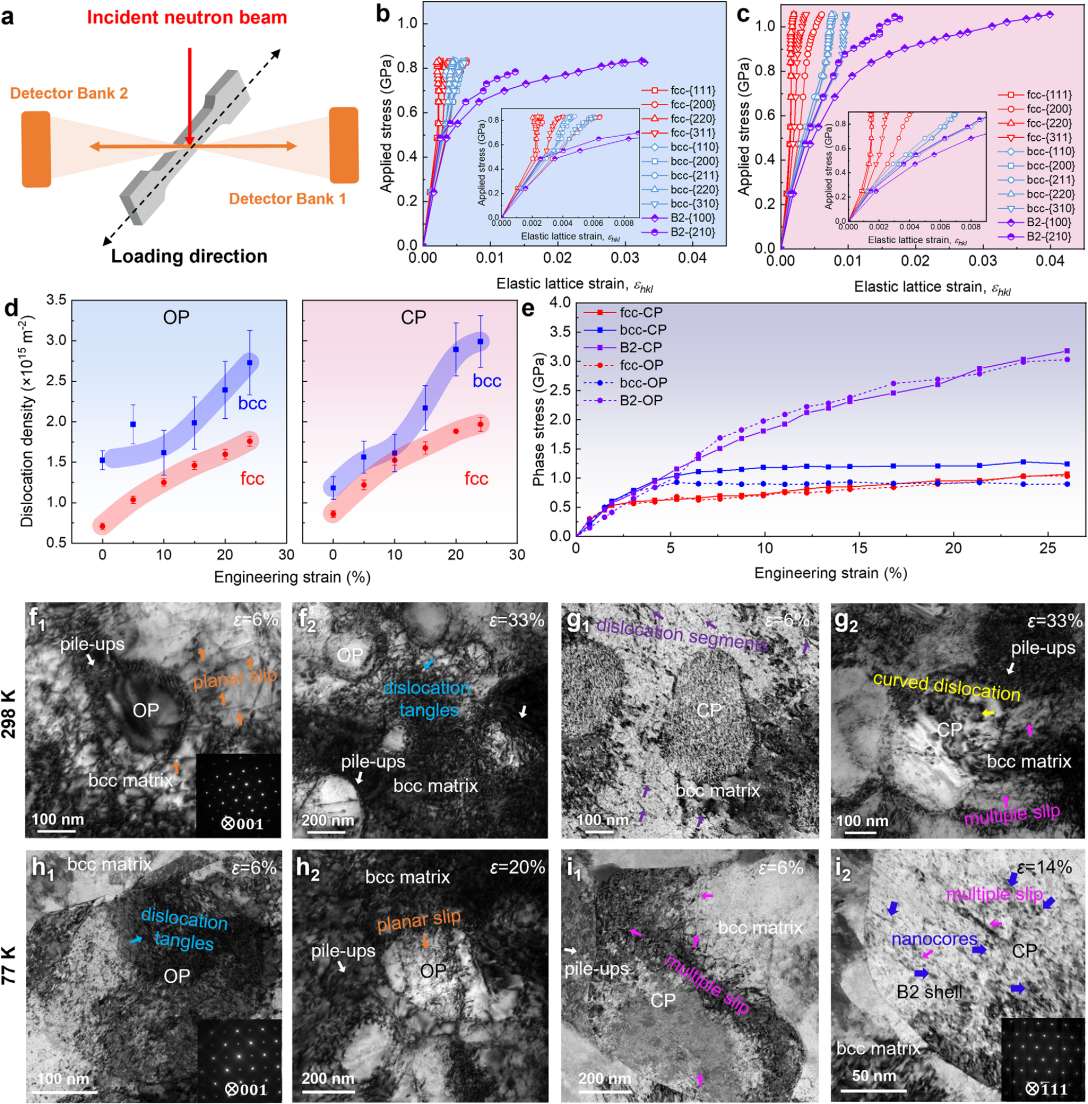
Deformation and substructures. (a) Schematic diagram of in situ neutron diffraction tensile testing set‐up. (b,c) Evolution of lattice strain against macroscopic true stress for representative fcc (including {111}, {200}, {220} and {311}), bcc (including {110}, {200}, {211}, {220} and {310}) and B2 (including {100} and {210}) crystallographic plane families along the loading direction in the OP and CP samples at 298 K, respectively. (d) Dislocation density against strains of the fcc and bcc phases in the OP and CP samples, respectively, calculated by the modified Williamson‐Hall method (Method). (e) Macroscopic stress‐strain response with the corresponding stress partitioning in the fcc, bcc, and B2 phases in the OP and CP samples. (f) The bright‐field TEM image of the bcc matrix in the OP sample at 298 K respective at small (f_1_) and large (f_2_) strains. (g) The bright‐field TEM image of the bcc matrix in the CP sample at 298 K respective at small (g_1_) and large (g_2_) strains. (h) The bright‐field TEM image of the bcc matrix in the OP sample at 77 K respective at small (h_1_) and large (h_2_) strains. (i) The bright‐field TEM image of the bcc matrix in the CP sample at 77 K respective at small (i_1_) and large (i_2_) strains.

The in situ neutron diffraction set‐up under tension is schematic in Figure 4a, where the diffraction signals can be detected both along the loading direction (LD, Bank 1) and transverse direction (TD, Bank 2). Figure 4b, c shows the progressive yielding and hardening behavior of fcc, bcc, and B2 phases along the LD in OP and CP samples, respectively. Both OP and CP samples exhibit essentially identical deformation behavior, and it should be noted that their deformation behaviors (including three stages) along LD and TD are consistent (see Figure S6). In stage I, before the yield point, the lattice strains respond proportionately to the applied stress. The {200} reflection exhibits the lowest elastic modulus, followed by {311}, {220} and {111} in the fcc matrix, meanwhile followed by {310}, {220}, and {211} in the bcc matrix. For the B2 MINPs, the {210} reflection is stiffer than {100} reflection. All these results consistent with the cubic elastic anisotropy factor [64, 65]. In stage II, a clear transition from linear to non‐linear point can be found, showing the onset of yielding as loads are redistributed. The lattice strain of the fcc‐{220} crystallographic family first deviates from linearity after the elastic deformation limits and turns upward. Meanwhile, other fcc reflections (especially {200} reflection) deviate from linearity but turn downward. Such a stiffening response is due to the load shedding from the {220} to other reflections [21]. When the applied stress increases, the progressive yielding of different oriented planes occurs sequentially, i.e., from fcc‐{220} to fcc‐{111}, fcc‐{311}, and then to fcc‐{200} reflections. The yielding/softening of the fcc matrix reflections is prior to that of the bcc matrix and the followed B2 MINPs reflections. By contrast, the lattice strain of bcc/B2 planes remains linear but the corresponding slope decreases with respect to the elastic stage, indicating a load transfer from the fcc matrix to the elastic bcc/B2 phases. Subsequently, the yielding of bcc‐{200} reflection triggers the co‐deformation of soft fcc and hard bcc matrix, leading to the micro‐yielding of the duplex laminated matrix. In stage III, both the B2 MINPs and the bcc matrix undergo strain hardening, since no increase in elastic lattice strains was observed with progressive deformation in fcc reflections, implying limited strain hardening of the fcc matrix.

Figure 4d reveals the dynamic evolution of dislocation density in the fcc and bcc matrix of OP and CP samples during plastic deformation, respectively. The initial dislocation density in the fcc matrix (*ρ*_fcc_) is consistently lower than that in the bcc matrix (*ρ*_bcc_), with the lowest *ρ*_fcc_ observed in the OP sample (∼7.1 × 10^14^ m^−2^). Throughout plastic deformation, *ρ*_fcc_ exhibits a stable and consistent increase of ∼1.0 × 10^15^ m^−2^, indicating a uniform dislocation multiplication mechanism in the fcc matrix. By contrast, *ρ*_bcc_ also shows a steady initial growth but diverges significantly in the CP sample, where it undergoes a sharp increase within the strain range of 15% to 20%, ultimately reaching ∼2.9 × 10^15^ m^−2^. Figure 4e illustrates the phase stress in the fcc, bcc, and B2 phases. The fcc matrix experiences the lowest stresses and significant strain hardening (stresses in the fcc matrix exceed the bcc matrix after strain of ∼20% in the OP sample), while expected higher interphase stresses in the bcc phase are observed in the CP sample. The geometric phase analysis (GPA) is also carried out in the structurally complex B2 MINPs after a slight tensile deformation (∼4%) of the CP sample. The nanocores are widely observed, exhibiting not only distortions in the disordered nanocores but also mobile dislocations with jagged advance in the B2 shell, see Figure S7.

At 298 K, dislocations in the bcc matrix of the OP sample exhibit planar slip and pile up against the ordinary B2 MINPs at small strains (Figure 4f, _1_), evolving into severely tangled configurations in the post‐mortem condition (Figure 4f_2_). In the CP sample, by contrast, dislocation segments appear within the bcc matrix at small strain (Figure 4g_1_), while at large strains multiple slip systems are activated in the bcc matrix and dislocations become curved within the core–shell B2 MINPs (Figure 4g_2_). The plastic deformation mechanisms of the B2 MINPs are even more complicated at 77 K. Figure 4h,i unveils the multiple slip becomes more dominated in the core–shell B2 MINPs. Meanwhile, the transformation of the dominant dislocations from <001>‐type (in the OP sample) to <111>‐type (in the CP sample) in the B2 MINPs (see more details in Figure S8). As for the fractured fcc matrix, no deformation twins (DTs) are activated in the OP sample with plastic deformation primarily governed by high‐density dislocation tangling unless the deformation temperature decreases to 77 K (Figure S9a,b). By contrast, at 298 K, the CP sample shows abundant nano‐DTs accompanied by high‐density stacking faults and immobile Lomer–Cottrell (L–C) locks (Figure S9c,d). The DTs‐dominant deformation mechanism of the fcc matrix is even more active at 77 K (Figure S9e,f).

## 3 Discussion

### 3.1 The Origin of the High Strength

At 298 K, compared to the CP sample although the OP sample contains B2 MINPs both in bcc and fcc matrix, it has relatively low *σ*_y_ combining similar *ε*_u_ (∼20%) and *ε*_t_ (∼33%). In the CP sample, the dislocation strengthening (Δσ_*d*_) of 303 MPa contribute most to the YS in the fcc matrix, followed by grain boundary (GB) strengthening (Δσ_*gb*_) of 133 MPa, solid solution strengthening including intrinsic friction stress (Δσ_*ss*_) 130 MPa and precipitation strengthening (Δσ_*p*_) 71 MPa. While in the bcc matrix, Δσ_*ss*_ ∼325 MPa contributes to the YS most, followed by Δσ_*d*_ ∼265 MPa, Δσ_*gb*_ ∼235 MPa and Δσ_*p*_ ∼93.5 MPa in a decending sequence. Note that the Δσ_*p*_ for both fcc and bcc phases is calculated based on the load‐bearing mechanism [66]. Load bearing is governed by the large (>200 nm), high‐modulus precipitates (the hard phase), which sustain most of the applied stress, while the softer matrix accommodates plastic strain through dislocation slip or twinning. Efficient stress transfer across strongly bonded interfaces from the matrix to the precipitates results in pronounced load partitioning and exceptional strength. As such, in the CP sample, the strengthening contribution is 637 and 919 MPa in the fcc and bcc matrixes, respectively; while in the OP sample, the strengthening contribution is 579 and 886 MPa in the fcc and bcc matrixes, respectively. More details about the strengthening contributions can be referred as to Note‐S3 and Table S5. By using the rule of mixtures (ROM), the estimated YS is ∼750 and ∼701 MPa in the CP and OP sample, respectively, based on the volume fractions of 40% and 60% for bcc and fcc phases, respectively. Note that the calculated strength (*σ*_cal_) is well consistent with the experimental strength (*σ*_exp_) of the OP sample. It indicates Type I back stress (overlapping with Δσ_*gb*_) dominates the hetero‐deformation‐induced (HDI) strengthening in the OP sample [67]. However, in the CP sample the *σ*_exp_ is higher than the *σ*_cal_. The enhanced yield strength of ∼100 MPa in the CP sample (equivalent to ∼250 MPa within the bcc matrix) originates primarily from the coherent core/shell interfaces and the chemical heterogeneities in the B2 shell. These features trigger Type II back stress, which augments HDI strengthening through an additional structural gradient hardening (SGH) mechanism [67], resulting in a markedly hindered, jogged advance of gliding dislocations (Figure S7). The downward of bcc/B2 reflections seem more pronounced in the CP sample (Figure 4c), also implying the extraordinary stress carrying capability of the B2 MINPs. The segregation of the Fe/Cr atoms in the nanocores not only enhance the elastic strain fields inside the MINPs created by the misfitting nanocores [68], but also promote the elastic interactions between the MINPs and dislocations in the matrix (the lattice misfit is much higher at the matrix/precipitate interfaces), contributing to the increased YS of CP samples.

At 77 K, the extra strengthening relative to the room‐temperature yield strength—approximately 450 MPa for the CP sample and 300 MPa for the OP sample (Note S3)—is attributed to the promotion of Type II back stress [67]. The structural gradient hardening established by the core/shell interfaces and the chemical heterogeneities in the B2 shell inevitably provides an additional ∼150 MPa of HDI strengthening. The soft fcc grains generally undergo the initial plastic deformation, and serious plastic‐strain gradients occur between the deformable (fcc) and nondeformable (bcc) grains, where the strength mismatch is ∼282 and ∼400 MPa at 298 and 77 K, respectively (Tables S5 and S6 and Figure S10a,b). These fcc grains cannot plastically deform freely due to the constraint by the elasticaly deformed bcc grains especially at 77 K. The hetero‐deformation between fcc and bcc grains will induce HDI stresses thereby strengthening these fcc grains [20]. In the bcc grains, the thermal energy available is insufficient to trigger dislocation slip overcoming the Peierls barrier and the elastic strain fields induced by the structurally complex B2 MINPs are further promoted and remained since the stress relaxation is significantly inhibited [69]. Both the HDI stress and the effective stress are improved (Figure S11), contributing to ultrahigh cryogenic YS.

### 3.2 The Origin of the Large Ductility

The CP alloys show a good strain hardening capacity, thus high UE at both temperatures. At 298 K, there are four possible factors contrtibute to the large ductility, as summarzed below. (i) In the fcc grains, apart from L–C locks, the DTs are activated to enhance the SHRs via the dynamic Hall‐Petch hardening effect when the local stress exceeds the critical twinning stress due to the incoherent MINPs in the CP sample (Figure S9c,d). (ii) In the bcc grains, the dislocation pile‐ups at the coherent interfaces would enhance the local strain concentrations, thus promote dislocation emission and multiplication. The notable increase of *ρ*_bcc_ (Figure 4d) and high SHRs are attributed to plenty of efficient dislocation emission sites caused by disordered nanocores. (iii) The reduced ordering energy (Figure S2b) improves the intrinsic ductility of the B2 MINPs [8]. The gliding dislocation lines become more tortuous because each segment responds differently as it meets chemical heterogeneities in B2, coined as “nanoscale segment detrapping” strengthening [70]. The progressive release of dislocation segments pinned at nanoscale obstacles, which generates sustained forest dislocation accumulation. The “stop and go” stick‐slip offers extra wait/dwell time to make the gliding dislocations more likely to hit stalled ones, and the wavy morphology increases the total line length and the chances for dislocations interactions. (iv) The HDI stress (Figure S11) increases with strains in the CP sample due to the profuse dislocation pile‐ups [71], implying the HDI strengthening also contributes to the high SHRs [72]. As a result, the structurally complex B2 MINPs enhance SHRs for excellent self‐hardening capacity. Numerous precipitate microcracking and deep dimples (Figure S10c,d) also demonstrate that the CP alloy has superior ductility and crack‐tolerant capacity.

At 77 K, the effect of the above four factors are amplified to allievate even avoid the cryogenic brittleness, and other factors also further enhance the SHRs. (i) In the bcc grain, a notable transformation of the dislocation slip occurs, i.e., the <111>‐type dislocations dominate the plastic deformation of MINPs at 77 K instead of the <100>‐type. This stands in contrast to the traditional B2 phase, where the CRSS of <111> slip can reach 500% of <100> slip [26, 27]. The multiple <111> dislocations enable higher ductility of the lattice compared to B2 MINPs where only <100> dislocations are activated [15]. (ii) High stresses stimulate the multiple slip of dislocations, facilitating dislocation interactions to inhibit slip softening for enhanced SHRs and large ductility at the cryogenic temperatures. It should be noticed that once a microcrack initiated in a hard B2 MINP with less effective in arresting cracks, it would rapidly run across which and eventually leading to premature failure (Figure S12 and Note‐S4) [9].

## 4 Conclusions

In summary, we have developed a duplex fcc/bcc Fe‐MEA whose strength–ductility combination at both 298 and 77 K surpasses that of conventional precipitate‐strengthened steels and multi‐principal‐element alloys. The coherent, structurally complex B2 MINPs in the bcc matrix and the incoherent ordinary B2 MINPs in the fcc matrix act synergistically to underpin coupled strengthening and ductilizing mechanisms, yielding an exceptional balance of high strength and large ductility. By harnessing a structural complexification strategy that confers self‐hardening capability on the B2 MINPs, we demonstrate a pathway to strength–ductility combinations that remain largely unmatched at both ambient and cryogenic temperatures. This work refines the fundamental understanding of phase transformations in precipitates and offers a design strategy of advanced alloys for cryogenic service.

## Author Contributions

J.Y.Z. designed the project and conceived the microstructural design strategy; J. S. guided the research; H.W. prepared the materials, S.H.G., X.X.F., S.Y.L., and J.L. conducted the mechanical properties, APT and AC‐TEM experiments, Y.Y. did the DFT calculations, W.L.S. did the in‐situ neutron diffraction experiments and analyzed the results; S.H.G. and J.Y.Z. wrote the corresponding text from the input of G.L. and H.W. All authors contribute to the discussion of results.

## Funding

This work was supported by the National Natural Science Foundation of China (Grant Nos. 52441407, 52431006, 92163201, U23A6013, 92360301, 52595631, U2330203, 523B2004, 52571016, U25B20115 and 52001184), the 111 project of China (B25007), Shaanxi Province (Youth) Innovation Team Project (2024RS‐CXTD‐58, 22JP042), the Shaanxi Provincial Natural Science Basic Research Plan (2025SYS‐SYSZD‐097), China Postdoctoral Science Foundation (GZB20250050), National Key R&D Program of China (2023YFB3711904), and the Fundamental Research Funds for the Central Universities (xtr062024006).

## Conflicts of Interest

The authors declare no conflicts of interest.

## Supporting information




**Supporting File**: advs75787‐sup‐0001‐SuppMat.docx.

## Data Availability

The data that support the findings of this study are available from the corresponding author upon reasonable request.
