# Supplementary material for: Structurally Complex Precipitates Enhance Strength‐Ductility Synergy in a Duplex Medium Entropy Alloy
Source: Adv Sci (Weinh). 2026 May 22;13(46):e75787. doi: 10.1002/advs.75787 (PMC13335870; doi:10.1002/advs.75787)
Supplement: Supplementary file 1 [file ADVS-13-e75787-s001.docx]

**Supporting Information for**

**Structurally Complex Precipitates Enhance Strength-Ductility Synergy in a Duplex Medium Entropy Alloy**

*Shaohua Gao, Yang Yang, Xiaoxuan Fan, Jinyu Zhang, * Shuaiyang Liu, Jiao Li, Hui Wang, * Wenli Song, * Gang Liu, and Jun Sun*

S. Gao, Y. Yang, X. Fan, J. Zhang, S. Liu, J. Li, G. Liu, J. Sun

State Key Laboratory for Mechanical Behavior of Materials, Xi’an Jiaotong University, Xi’an, 710049, PR China

H. Wang

College of Materials Science and Engineering, Sichuan University, Chengdu, 610065, PR China

W. Song

Institute of High Energy Physics, Chinese Academy of Sciences (CAS), Beijing, 100049, PR China

W. Song

Spallation Neutron Source Science Center, Dongguan, 523803, PR China

Email*:* [jinyuzhang1002@xjtu.edu.cn](mailto:jinyuzhang1002@xjtu.edu.cn); wanghui1984@scu.edu.cn; [songwl@ihep.ac.cn](mailto:songwl@ihep.ac.cn)

**The file includes:**

**Materials and Methods**

**Notes. S1-S4**

**Figs. S1-S12**

**Tables. S1-S7**

**Supporting Information References**

# Materials and Methods

**Sample fabrication.**

The bulk Fe_58_Ni_16_Cr_16_Al_10_ (at%) duplex Fe-MEA ingot was prepared using commercial metals (purity >99.95 wt %) by vacuum induction melting under Ar atmosphere. The ingot was re-melted at least five times to ensure the chemical homogeneity. The as-cast alloy was hot-rolled to a 70% reduction at 1100–1200 °C, followed by water quenching (hereafter referred to as the initial sample). The initial samples were subsequently annealed under three distinct conditions: 1200 °C for 30 min, 900 °C for 1 h, and 800 °C for 1 h, producing the DL (duplex heterogeneous micro-lamellar), OP (strengthened by ordinary multicomponent intermetallic nanoprecipitates), and CP (strengthened by complex multicomponent intermetallic nanoprecipitates) samples, respectively. The DL sample was water-quenched, whereas the OP and CP samples were air-cooled.

**Experimental methodology.**

The microstructure of precipitation in the duplex matrix was observed using scanning electron microscope (SEM, Zeiss Sigma 300). Electron back-scattered diffraction (EBSD) maps were conducted on a Gemini-SEM 500 instrument at step size of 350 nm. All samples were mechanically polished first and finally electro-polished using 10% perchloric acid in ethanol at a voltage of ~20 V. The selected micro-zones of the OP and CP samples are of the same size and the acquisition steps are the same.

To study the microstructures at nanoscale, transmission electron microscope (TEM, JEM-F200) was employed, and spherical-aberration-corrected STEM (JEM-ARM200F) was used to perform HAADF-STEM and energy dispersive spectroscopy (EDS) maps, operated at 200 kV. The TEM foils and APT tip specimens were prepared using a Carl Zeiss Auriga FIB scanning electron microscope (SEM). APT measurements were performed using a local electrode atom probe (CAMECA LEAP 4000X Si) with an ultraviolet (UV) laser pulse repetition rate of 200 kHz, at a pulsing laser energy of 60 pJ, a target evaporation rate of 0.5%, and a specimen temperature of 20 K. The results were reconstructed and analyzed using CAMECA’s interactive visualization and analysis software (IVAS 3.6.8).

Tensile and loading-unloading-reloading (LUR) tests tests were conducted at ambient/cryogenic temperature at an initial strain rate of 10^-3^ s^-1^ by a Kammrath & Weiss tensile stage, using the dog-bone shaped specimen with a gauge length of 10 mm, a width of 4 mm, and a thickness of 1.5 mm, with their longitudinal axes parallel to the rolling direction (RD). Note that the cryogenic tensile samples were immersed in liquid nitrogen during the whole tensile process (soak for 20 min to ensure thermal equilibrium and consistency before tensile tests). Single edge bend (SE(B)) specimens were cut with a thickness (B) of 3 mm, a width (W) of 7 mm (1≤W/B≤4), a span distance (S) of 28 mm (S/W=4) and total length of 32 mm meanwhile the longitudinal axes parallel to the RD. A notch (~3 mm in length) was cut and fatigue pre-cracking was performed to a total original crack length of 3.15~3.85 mm (The crack size, total length of the starter notch plus the fatigue crack, shall be between 0.45W and 0.55W, which is 3.48 mm). See more calculation details of the *J-*integral curves in Note-S2.

Neutron data were collected continuously during the tensile tests from two banks of detectors aligned with diffraction directions parallel and perpendicular to the loading direction. Diffraction from a subset of grains within the gauge volume that have the crystal lattice planes with normal (*hkl* in cubic crystal systems) parallel or perpendicular to load direction and satisfies Bragg’s law will be detected. To simplify, only the parallel bank was considered. The diffraction data was later chopped into 20-min segments to collect sufficient neutron counts to quantify fcc/bcc matrix and B2 precipitates lattice strain. The discussed lattice strain $\varepsilon_{hkl}$; defined by the equation: $\varepsilon_{hkl}=\left( d_{hkl}-d_{0,hkl} \right)/{d_{0,hkl}}$; where $d_{hkl}$ is the lattice interplanar spacing and $d_{0,hkl}$ is a reference lattice interplanar spacing collected with a negative small load applied. Individual diffraction peaks from the neutron spectra were fitted by single-peak fitting using a "Pseudo-Voigt" function to determine the peaks position and full width at half maximum (FWHM). Error for all data was determined automatically by the GSAS peak fitting software, and it often was small enough that it is invisible in the plots. In some cases, there were two distinct peaks with very similar *d*-spacings that were fit as individual, overlapping peaks, for instance, the bcc-{110} peak was predicted to overlap with the B2-{110} peak, but the intensity of the B2 peak was too low to observe it individually; the B2 peak was assumed to not contribute to the total intensity, peak position and FWHM of the two overlapping peaks. Note that any measurements of lattice strains and FWHM in the B2 precipitates include those precipitates in the fcc grains and bcc grains. Note that the actual FWHM of the sample is not equal to the measured value, and the peak width caused by the instrument itself needs to be removed with a standard sample.

**First principal theory calculations.**

We calculated the chemical ordering energy via Ordering Energy = *E*_ordered_ - *E*_random_, where *E*_ordered_ and *E*_random_ are the total energies of chemically ordered and random-solution structures containing the same constituent species.^[^^1^^]^ The DFT calculations are implemented using the Vienna Ab initio Simulation Package (VASP).^[^^2^^,^ ^3^^]^ The Coulomb interaction of ion cores with the valence electrons and the electronic exchange were described by Projector-augmented wave potential and the Perdew Burke-Ernzerhof generalized gradient approximation, respectively.^[^^4^^]^ For fcc structures, we use supercells with 3×3×3 unit cells (108 atoms in total). For the B2 supercells, we use 4×4×4 unit cells (128 atoms in total) to determine the total energy by using the 4×4×4 K-points with a plane-wave energy cutoff of 300 eV. Equilibrium cell volumes and internal atomic positions of the supercells were fully relaxed until the total energy differences < 10^-6^ eV. The models were generated by a special quasirandom structure (SQS) method via RMC Profile.^[^^5^^]^

**Dislocation densities calculations.**

The dislocation densities were calculated using the modified Williamson-Hall (MWH) method, quantifying the line broadening as follows:^[^^6^^]^

$\left( \Delta K \right)^{2}=\left( 0.9/D \right)^{2}+\left( \pi A^{2}b^{2}\rho/2 \right)\left( K^{2}\overline{C} \right)+O\left( K^{2}\overline{C} \right)^{2}$,	(S1)

where *K=1/d*, *ΔK=-K(Δd/d)*, *d* is the interplanar spacing and *Δd* is the full width at half maximum (FWHM) of the diffraction peaks, *b* is the Burgers vector length of dislocation, *ρ* is the dislocation density. Note that the actual FWHM of the sample is not equal to the measured value, and the peak width caused by the instrument itself needs to be removed with a standard sample. Strain anisotropy is rationalized by the dislocation contrast factor *C_hkl_*. The average $\overline{C}$ can be determined by $\overline{C}=\overline{C}_{h00}\left( 1-qH^{2} \right)$, where *H^2^=(h^2^k^2^+ h^2^l^2^+ k^2^l^2^)/(h^2^+ k^2^+ l^2^)^2^*, and $\overline{C}_{h00}$ is a constant depending on the dislocation type (*q* values: edge or screw) and anisotropic elastic constants *C_11_*, *C_12_* and *C_44_*: 198, 125 and 122 GPa in fcc; 231, 134 and 116 GPa in bcc, respectively.^[^^7^^]^ *A* is a constant depending on the effective outer cutoff radius of dislocations, *A_i_=2C_44_/(C_11_- C_12_)*. In our case of dislocations,^[^^8^^]^ $\overline{C}_{h00}=0.1740\left[ 1-\exp\left( -\frac{A_{i}}{1.9522} \right) \right]+0.0293A_{i}+0.0622$ in bcc where *C_12_/C_44_≈*1, $\overline{C}_{h00}=0.1687\left[ 1-\exp\left( -\frac{A_{i}}{2.0400} \right) \right]+0.0194A_{i}+0.0926$ in fcc where *C_12_/C_44_≈*1; and $q=8.6590\left[ 1-\exp\left( -\frac{A_{i}}{0.3730} \right) \right]+0.0424A_{i}-6.074$ in bcc where *C_12_/C_44_≈*1, $q=4.8608\left[ 1-\exp\left( -\frac{A_{i}}{0.8687} \right) \right]+0.0896A_{i}-3.4280$ in fcc where *C_12_/C_44_≈*1. Accordingly, the slope of the MWH plot, $\left( \Delta K \right)^{2}$ versus $K^{2}\overline{C}$ gives the $\pi A^{2}b^{2}\rho/2$, then we get $\rho$ in bcc and fcc at different tensile strains.

**Interphase stress partitioning between the fcc and bcc phases.**

The stress partitioning between the fcc and bcc matrix can be calculated by analyzing the evolution of lattice strains along axial and transverse directions from the *in-situ* neutron diffraction peaks as follows:^[^^9^^,^ ^10^^]^

$\sigma_{11}=\frac{E}{1+\nu}\varepsilon_{11}+\frac{\nu E}{\left( 1+\nu\right)\left( 1-2\nu\right)}\left( \varepsilon_{11}+\varepsilon_{22}+\varepsilon_{33} \right)$,	(S2)

$\sigma_{22}=\sigma_{33}=\frac{E}{1+\nu}\varepsilon_{22}+\frac{\nu E}{\left( 1+\nu\right)\left( 1-2\nu\right)}\left( \varepsilon_{11}+\varepsilon_{22}+\varepsilon_{33} \right)$,	(S3)

where $\sigma_{11}$ is the principal axial stress, and $\sigma_{22}$ and $\sigma_{33}$ (assuming $\sigma_{22}=\sigma_{33}=0$ for simplification) are the principal transverse stresses. $E$ is the elastic modulus of the {311} (257 GPa for CP and 308 GPa for OP) and {211} (162 GPa for CP and 138 GPa for OP) planes for fcc and bcc (including B2) phases, respectively, because that stress is less affected by the intergranular stress, and the Poisson’s ratio $\nu$ is the same. $\varepsilon_{22}$ and $\varepsilon_{33}$ are the measured lattice strains for the axial and transverse directions (where $\varepsilon_{22}$ and $\varepsilon_{33}$are equal). As a result, the phase stresses can be estimated from the dynamic evolutions of *in-situ* neutron diffraction peaks.

**Note-S1 The Formation for Structurally Complex B2 MINPs in the Duplex Matrix.**

During hot-rolling (HR), the bcc phase serves as the primary matrix, from which the fcc phase precipitates; the grains of both phases recrystallize and align along the longitudinal direction of the bar, forming a micro-duplex lamellar structure.^[^^11^^]^ This duplex structure is retained upon cooling from the high-temperature two-phase field to the room-temperature two-phase field, aided by the addition of Ni, which acts as an fcc austenite stabilizer.

As a feasible strategy for achieving the special microstructure in ordered B2 precipitates, we can take advantage of diffusion-controlled phase transformation. However, this strategy encounters two challenges: (i) It is quite difficult to select the alloying solutes content and corresponding heat-treatment temperature in a typical NiAl-B2 precipitates. (ii) In order to effectively alleviate the strain localization, the nanocores phase should be fine and homogenous, preferably forming a dispersed cores in shell microstructure.

In Al_0.5_CoCrFeNi alloy,^[^^12^^]^ first-principles calculations and Monte-Carlo simulations have revealed Ni and Al are assumed to form the basis of the two sublattices. Both Cr and Fe solutes can easily occupy the sublattice of Al (preferable) or Ni, while Fe solutes are lazier than Cr to occupy them.^[^^12^^]^ It is comforting to see that the changes in the first nearest neighbor bonding of Fe-Cr do not increase or decrease, indicating that FeCr-rich bcc phase is more of a disordered solid solution.^[^^12^^]^ An in-situ heating TEM observation reveals that Cr is a substitutional element during post-annealing treatment in a multicomponent hierarchical precipitate.^[^^13^^]^

In our Fe-Ni-Cr-Al system, Cr atoms, characterized as fast-diffusion solutes within the bcc matrix (Figure S1a). The formation energy (Figure S2a) and ordering energy (Figure S2b) of the bcc matrix, sphere B2 precipitates and nanocores were estimated using DFT, based on the compositions derived from APT (Figure 2). The sphere B2 phase exhibits the strongest atomic bonding, with an ordering energy of approximately -0.18 eV per atom (Figure S2b), significantly stronger than that of the bcc matrix (-0.006 eV per atom), followed by the preferential formation of disordered nanocores (Figure S2b). The strong Ni-Al bonding in the initially homogeneous solid solution at elevated temperatures drives the formation of a substantial number of Ni-Al atomic pairs during cooling, attributed to the greatest negative mixing enthalpy between Ni and Al atoms (see Table S1). As these pairs aggregate, the B2 phase begins to nucleate while Cr solutes readily enrich around the nuclei of the B2 precipitates (Figure S2c), inducing pronounced chemical heterogeneities. Analogous to the formation of the cores in core-shell Al_3_(Sc, Zr) nanoparticles in Al-Sc-Zr alloys, where fast-diffusion Sc atoms tend to aggregate inside the particles.^[^^14^^,^ ^15^^]^ Note that Sc atoms are consumed by Al_3_Sc precipitation, while Cr is not the forming element of B2 precipitates. These Cr solutes bear a similar behavior to Mg atoms in Al-Mg-Sc alloys,^[^^16^^]^ where the rapid diffusion and segregation of Mg solutes lead to the formation of Al_3_(Mg, Sc)_2_ nanoparticles exhibiting a Mg-shell structure. In our case, Cr atoms in the Cr-rich bcc matrix diffuse toward the edge of the Cr-poor B2 phase, preferentially through a downhill diffusion mechanism. This ordered-disordered transformation is attributed to the increasing Cr content (Figure S2b), driven by a diffusion-dominated process.

It is desirable, therefore, to devise novel internal homogeneous nanostructures in intrinsically brittle particles. Recent developments have shown that the core-shell structured carbides are formed via interphase precipitation, where the kinetics of the cores depends on the balance between its driving force and the mismatch with the surrounding matrix.^[^^17^^,^ ^18^^]^ Since the lattice parameter of the large precipitate is greater than that of the matrix, and a flux of constitutional vacancies in NiAl-B2 precipitates^[^^19^^]^ (provided by hot deformation process) is required to accommodate the internal stresses arising from the growth of precipitates.^[^^20^^]^ The vacancy diffusion is happening; thus, high temperature will not only increase the entropy term but also facilitate diffusion mismatch, resulting significant chemical heterogeneities within the ordered phase. The chemical heterogeneities within the B2 phase is initiated by the trapping of Cr atoms and subsequently amplified through diffusion-controlled coarsening assisted by the constitutional vacancies in NiAl-B2 precipitates (Figure S2c).^[^^19^^]^ Eventually, the Cr-rich nanocores undergo a spinodal decomposition process,^[^^21^^,^ ^22^^]^ driven by the uphill diffusion of Cr atoms (Figure S2c), as evidenced by the broad diffusion interface observed at the core-shell boundary (Figure S1b). In short, the incorporation of Cr into the B2 phase can be explained by two distinct mechanisms: (i) the capture of Cr atoms during the nucleation of the B2 phase, and (ii) the consumption of Cr during the growth of the B2 phase from the bcc matrix. These dual mechanisms are further confirmed by the abundant and dispersed nature of the core nanostructures. By contrast, rod-shaped B2 precipitates within the fcc matrix lack Cr-rich nanocores, a consequence of the slower diffusion kinetics of Cr in the fcc structure and the inherently lower Cr content in the fcc matrix, which collectively suppress both nucleation and gradient diffusion mechanisms.

**Note-S2 The Fracture Toughness Calculation and Comparison**

Given their superior damage tolerance, we investigated the room-temperature fracture toughness of the OP and CP samples by performing nonlinear *J*-based fracture toughness tests (see Methods). Full resistance-curve (*R*-curves) measurements that reveal both the crack-initiation and crack-growth fracture toughness are shown in Figure. S5a. Single edge bend (SE(B)) specimens were cut by electric discharge machine with a thickness (*B*) of 1.75 mm, a width (*W*) of 7 mm (1≤*W/B*≤4), a span distance (*S*) of 28 mm (*S/W*=4) and total length of 32 mm meanwhile the longitudinal axes parallel to the rolling direction. A notch (~3 mm in length) was cut and fatigue pre-cracking was performed on all specimens at room temperature to a total original crack length of 3.15~3.85 mm. The subsequent subcritical cracks growth was determined using the unloading compliance method according to the *ASTM* standard E1820.^[^^23^^]^ The instantaneous crack length, *a_i_* of the SE(B) specimens was determined from a resistance curve test method at the notched edge:^[^^23^^]^

${a_{i}}/W=0.999748-3.9504\mu+2.9821\mu^{2}-3.21408\mu^{3}+51.51564\mu^{4}-113.031\mu^{5}$(S4)

μ = $\frac{1}{{(BEC_{(i)})}^{1/2} +1}$	(S5)

where *C_(i)_* is the elastic unloading slope. For each crack length *a_i_*, the *J_i_*-integral was calculated as the sum of the elastic *J_el(i)_* and plastic *J_pl(i)_* components:

$J_{i}= J_{el(i)}+J_{pl(i)}= \frac{K_{i}^{2}}{E^{'}}+J_{pl(i)}$	(S6)

where *E′* = *E* (Young’s modulus) in plane stress or *E*/(1 - *ν*^2^) in plane strain (*ν* is Poissons ratio). For the present alloy, *E* is 180 GPa detected from the tensile curves, while *ν* is 0.3. The linear elastic stress intensity *K_i_* was defined for SE(B) specimen from:

$K_{i}= \frac{P_{i}}{{(BB_{N}W)}^{1/2}}$ $f\left( \frac{a_{i}}{W} \right)$	(S7)

where *P_i_* is the applied load prior to the partial unloading, *B* and *B_N_* are the specimen thickness and the net side-grooved thickness, respectively.

$f\left( \frac{a_{i}}{W} \right)=\frac{3\left( \frac{a_{i}}{W} \right)^{1/2}\left[ 1.99-\left( \frac{a_{i}}{W} \right)\left( 1-\frac{a_{i}}{W} \right)\left( 2.15-3.93\left( \frac{a_{i}}{W} \right)+2.7\left( \frac{a_{i}}{W} \right)^{2} \right) \right]}{2\left( 1+2\frac{a_{i}}{W} \right)\left( 1-\frac{a_{i}}{W} \right)^{3/2}}$	(S8)

The $f\left( \frac{a_{i}}{W} \right)$ is a geometry factor determined by the ratio of the crack length to the specimen width. For advancing cracks, the crack-growth corrected plastic component of $J_{i}^{\text{pl}}$ was evaluated from the following equation:^[^^23^^]^

$J_{i}^{\text{pl}}=\left[ J_{i-1}^{\text{pl}}+\left( \frac{\eta_{pl(i-1)}}{b_{i-1}} \right)\frac{A_{i}^{\text{pl}}-A_{i-1}^{\text{pl}}}{B_{N}} \right]\left( 1-\gamma_{pl(i-1)}\frac{a_{i}-a_{i-1}}{b_{i-1}} \right)$	(S9)

$A_{i}^{\text{pl}}-A_{i-1}^{\text{pl}}=\frac{\left( P_{i}+P_{i-1} \right)\left( \nu_{i}^{\text{pl}}-\nu_{i-1}^{\text{pl}} \right)}{2}$	(S10)

where $\eta_{pl(i-1)}=3.667-2.199\left( \frac{a_{\left( i-1 \right)}}{W} \right)+0.437\left( \frac{a_{\left( i-1 \right)}}{W} \right)^{2}$ , $\gamma_{pl\left( i-1 \right)}=0.131+2.131\left( \frac{a_{\left( i-1 \right)}}{W} \right)-1.465\left( \frac{a_{\left( i-1 \right)}}{W} \right)^{2}$and $b_{i}=W-a_{i}$ is the uncracked ligament length and $\nu_{i}^{\text{pl}}=v_{i}-\left( P_{i}C_{\left( i \right)} \right)$ is the plastic part of the crack mouth opening displacement. The quantity $A_{i}^{\text{pl}}-A_{i-1}^{\text{pl}}$ is the increment of the plastic work A^pl^ under the load vs. the crack mouth opening displacement curve. Using Eqs. (S4) to (S10), we can construct the J-Δa resistance curve by calculating the J_i_-integral corresponding to the crack extension Δa_i_.

The provisional toughness *J_Q_* was determined as the intersection of the *R*-curve and the 0.2 mm offset/blunting line with a slope of 2$\sigma_{n}$, where $\sigma_{n}=0.5\left( \sigma_{y}+\sigma_{UTS} \right)$ is the flow stress (referred to as the effective yield strength in the *ASTM* standard 1820 ^[^^23^^]^) as the average value of the yield strength and the ultimate tensile strength. In this work, the plane strain condition is well satisfied and the *J_Q_* to be considered as a size-independent fracture toughness (*J_Ic_*). Then, *K_JIc_* can be obtained from *J_Ic_*, using $K_{JIc}=\sqrt{E^{'}J_{Ic}}$, *E′* = *E*/(1 - *ν*^2^). The *J_Q_* values satisfy the specimen size requirements (*b_0_*, *B* > 10*J_Q_*/*σ_n_*) for both *J*-field dominance and plane-strain conditions, therefore they can be regarded as the valid *J_Ic_* and *K_JIc_* values.

The *K_JIc_* (defined at the 0.2 mm crack extension) and *K_SS_* (defined at the 1 mm crack extension) values of the CP sample are, respectively, 173.7 MPa·m^0.5^ and 210.1 MPa·m^0.5^, whereas the corresponding values for the OP sample are 188.3 MPa·m^0.5^ and 250.0 MPa·m^0.5^. The CP sample exhibits a considerable damage tolerance, further, we compared the fracture toughness *vs*. yield strength combination with other alloys as shown in Figure S5b, *e.g.*, medium Mn steels,^[^^24^^]^ high C steels,^[^^25^^]^ bainite steels,^[^^26^^]^ martensitic (stainless) steels,^[^^27-29^^]^ maraging steel,^[^^30^^]^ TRIP steels,^[^^31-33^^]^ duplex (stainless) steels,^[^^34^^]^ and high/medium entropy alloys (M/HEAs).^[^^35-37^^]^

**Note-S3 The Calculation of Strengthening Contributions**

The yield strength at 298 K of the CP sample can be obtained as a sum of the four individual contributions: solid solution strengthening including intrinsic friction stress ($\Delta\sigma_{ss}$), grain boundary strengthening ($\Delta\sigma_{gb}$), dislocation strengthening ($\Delta\sigma_{d}$), and precipitation strengthening ($\Delta\sigma_{p}$).

The solid solution strengthening was predicted by Varvenne’s SSH model, where the concept of misfit volume $\Delta V_{n}$ is critical, implying the local lattice distortion.^[^^38^^,^ ^39^^]^ Thus, the $\Delta V_{n}$ of each specific solute that reflects the average local atom environment around the element can be expressed as:^[^^40^^,^ ^41^^]^

$\Delta V_{n}=\sum_{m} c_{m}\left[ \frac{\partial\overline{V}}{\partial c_{n}}-\frac{\partial\overline{V}}{\partial c_{m}} \right],$	(S11)

where *c_m_* and *c_n_* represent the concentration of the solute-*m* and solute-*n* species, respectively. For the sake of simplicity, $\overline{V}\text{=}\sum_{n} c_{n}V_{n}$, $\Delta V_{n}=V_{n}-\bar{V}$.^[^^38^^,^ ^39^^]^ Varvenne et al ^[^^38^^]^ have confirmed the reliability of the $V_{n}$ values including Ni (10.94 Å^3^), Cr (12.27 Å^3^), Fe (12.09 Å^3^), and Al (14.0 Å^3^) in Cantor fcc alloy family. Here, $V_{n}={\left( \frac{4}{\sqrt{2}}r_{n} \right)^{3}}/4$ in FCC and $V_{n}={\left( \frac{4}{\sqrt{3}}r_{n} \right)^{3}}/2$ in bcc, where $r_{n}$ is the atomic radius. Then we deduce the $V_{n}$ values in bcc, including Ni (11.91 Å^3^), Cr (13.36 Å^3^), Fe (13.16 Å^3^), and Al (15.24 Å^3^). The predicted zero-temperature, shear yield stress $\tau_{y0}$ and energy barrier $\Delta E_{b}$ for dislocation gliding thermally activated can be written as:^[^^38^^,^ ^39^^]^

$\tau_{y0}=A_{\tau}\alpha^{-\frac{1}{3}}\mu\left[ \frac{1+\nu}{1-\nu} \right]^{\frac{4}{3}}\left[ \frac{\sum_{n} c_{n}\Delta V_{n}^{2}}{b^{6}} \right]^{\frac{2}{3}},$	(S12)

and

$\Delta E_{b}=A_{E}\alpha^{\frac{1}{3}}\mu b^{3}\left[ \frac{1+\nu}{1-\nu} \right]^{\frac{2}{3}}\left[ \frac{\sum_{n} c_{n}\Delta V_{n}^{2}}{b^{6}} \right]^{\frac{1}{3}},$	(S13)

where the dissociated fcc edge dislocation and the undissociated bcc edge dislocation lead to accurate alloy independent factors ($A_{\tau},A_{E}$)=(0.01785, 1.5618) for fcc alloy ^[^^38^^]^ and ($A_{\tau},A_{E}$)=(0.040, 2.00) for bcc alloys.^[^^42^^]^ Here, $\mu$ is the shear modulus (~75 GPa for fcc and ~80 GPa for bcc^[^^43^^]^) and $b$ is the Burgers vector of the dislocation (0.255 nm for fcc and 0.249 nm for bcc^[^^44^^]^). Additionally, this model involves with α = 0*.*125 (fcc) or 0.0833 (bcc) to the edge dislocation line tension as *Γ* = α*μb*^2^.^[^^45^^]^ At finite temperature $T$ and finite strain rate $\dot{\epsilon}$, standard thermal activation theory then leads to the predicted tensile yield stress as:

${\Delta\sigma}_{ss}\left( T,\dot{\epsilon} \right)=M\tau_{y0}\left[ 1-\left( \frac{kT}{\Delta E_{b}}\ln\frac{\dot{\epsilon_{0}}}{\dot{\epsilon}} \right)^{\frac{2}{3}} \right].$	(S14)

where $M$ is the Taylor factor (~3.06 for fcc and ~2.95 for bcc^[^^43^^]^) $\dot{\epsilon}$= 10^-3^ s^-1^ is the experimental strain rate, $\dot{\epsilon_{0}}$= 10^4^ s^-1 [^^38^^]^ is a reference strain rate, and k = 1.38 × 10^-23^ J·K^-1^ is Boltzmann constant. The composition is Ni_12_Al_7_Fe_65_Cr_16_ (at%) in the fcc matrix and Ni_3_Al_6_Fe_68_Cr_23_ (at%) in the bcc matrix. Based on Eqs. (S11)-(S14), we can obtain the predicted ${\Delta\sigma}_{ss}$ = 130 MPa for the fcc matrix, while ${\Delta\sigma}_{ss}$ = 325 MPa for the bcc matrix at 298 K in the CP sample.

The grain boundary strengthening follows the Hall-Petch (H-P) relationship:

$\Delta\sigma_{gb}=k_{s}/\sqrt{d}$,	(S15)

where $k_{s}$ is the H-P constant for dislocation slip (~14.55 MPa·mm^0.5^ in the fcc phase and ~18.97 MPa·mm^0.5^ in the bcc phase^[^^46^^]^) and $d$ is the average grain diameter. The contribution of $\Delta\sigma_{gb}$ is 133 MPa (at $d$ ~12 μm) in the fcc matrix and 235 MPa (at $d$ ~6.5 μm) in the bcc matrix, respectively.

The dislocations hardening effect is estimated by the Taylor hardening law:

${\Delta\sigma}_{d}=M\alpha\mu b\sqrt{\rho_{d}}$	(S16)

where $\alpha$ is a constant ~0.2.^[^^43^^,^ ^47^^]^ The densities of geometrically necessary dislocations (GNDs) can be roughly estimated from KAM value using the simple formula:^[^^48^^]^ $\rho_{GND}=\frac{2KAM}{\varphi b}$, where $\varphi$ is the acquisition step size. The GNDs density can be calculated on the order of 5.0×10^14^ m^-2^ and 3.8×10^14^ m^-2^ for fcc and bcc phases, respectively, based on the EBSD results. The total dislocation densities $\rho_{d}$ (including statistically stored dislocations, which is often on the order of 15% ~ 35% of the total dislocation density under the annealing condition^[^^49^^]^) is 6.7×10^14^ m^-2^ for the fcc matrix and 5.1×10^14^ m^-2^ for the bcc matrix. Accordingly, the contribution of ${\Delta\sigma}_{d}$ is 303 and 265 MPa in fcc and bcc matrices, respectively.

To quantify the contribution of precipitation strengthening mechanism considering of the precipitation size, the load-bearing mechanism is expressed by the following equation:^[^^50^^]^

$\Delta\sigma_{p}=0.5V_{f}\sigma_{y}$,	(S17)

where $V_{f}$ and $\sigma_{y}$ is the volume fraction of the precipitates and yield strength of the sample, respectively. For the rod MINPs in the fcc matrix, in the CP sample ($\sigma_{y}$=856 MPa), the rod MINPs have $V_{f}$= 16.6%, and ${\Delta\sigma}_{p}$=71 MPa. For the sphere MINPs in the bcc matrix, the contribution of ${\Delta\sigma}_{p}$ is 93.5 MPa with $V_{f}$= 21.9%.

As for the OP sample, we just consider the difference of the strengthening contributions of $\Delta\sigma_{gb}$, $\Delta\sigma_{d}$ and $\Delta\sigma_{p}$ at 298 K. In the fcc constituent, these three factors are 118, 287 and 43.8 MPa, respective. In the bcc constituent, these three factors are 239, 271 and 50.5 MPa, respective. Considering the volume fraction of the bcc (40%) and fcc (60%), the rule of mixtures (ROM) was employed to estimate the yield strength both in the CP and OP sample (see more details in Table S5). The calculated yield strength is ~750 MPa and ~700 MPa respective in the CP and OP sample at 298 K.

As for CP sample at 77 K, the $\Delta\sigma_{ss}$ equals to 213 MPa in the fcc matrix and 529 MPa in the bcc matrix. The increasing of $\Delta\sigma_{gb}$ is nearly unaffected by temperature.^[^^51^^]^ As for $\Delta\sigma_{d}$, the thermal activation contributions are affected by the shear modulus changes with temperature *T* as *μ*=*μ*_RT_[1-0.5(*T*-300)/*T_m_*], where *μ*_RT_ is the shear modulus in room temperature and *T_m_* is the melting point (1700 K).^[^^52^^]^ Then, the shear modulus at 77 K (*μ*_CT_) is 80 GPa in the fcc matrix and 85 GPa in the bcc matrix. The contributions of yield strength are 323 MPa ($\Delta\sigma_{d}$) in the fcc matrix, and 282 MPa ($\Delta\sigma_{d}$) in the bcc matrix, respectively. Assuming $\Delta\sigma_{p}$ (load-bearing mechanism) remains unchanged, the calculations of yield strength are 740 MPa in fcc and 1139.5 MPa in bcc, respectively. Based on the volume fraction of the fcc (60%) and bcc (40%), the eventual calculation of yield strength is ~900 MPa (see Table S6). The calculated strength is much lower (~450 MPa) than the experimental strength. For the OP sample at 77 K, $\Delta\sigma_{ss}$, $\Delta\sigma_{gb}$, $\Delta\sigma_{d}$, and $\Delta\sigma_{p}$ are 213/529, 118/239, 306/289 and 43.8/50.5 MPa in the fcc/bcc matrix, respectively, see more details in Table S6 (Supporting Information). The calculated yield strength via the rule of mixtures (ROM) is 851 MPa, which is ~300 MPa lower than the experimental value (1150 MPa). This difference indicates a weaker HDI strengthening effect in the OP sample compared with the CP sample.

**Note-S4 The Microcracking Behavior**

To elucidate the mechanism responsible for the ductility reduction at 77 K, we further examined the fracture surfaces. As shown in revised Fig. S12, conspicuous cracks initiated at and propagated across the spherical B2 MINPs embedded in the fcc matrix at 77 K. On the one hand, the ductile matrix surrounding the B2 crack tips can act as an efficient crack arrester, suppressing further crack propagation into the matrix. On the other hand, the interfaces between the fcc matrix and the spherical B2 MINPs without the core–shell structure are not fully coherent. Where slip transmission across the interface is discontinuous, dislocations pile up against the interface, generating severe stress concentrations. With decreasing temperature, these local stress concentrations are dramatically amplified by the elevated applied shear stresses, as described by the double-ended dislocation pile-up model^[^^53^^]^ *i.e.*, $\tau_{local}=N\tau_{a}=\frac{(1-\nu){{l\tau}_{a}}^{2}}{\mu b}$ and $l=\frac{\mu^{2}b}{{\sigma_{y}}^{2}}$ ^[^^54^^,^ ^55^^]^ where $N$ is the number of dislocations in a pile-up, $\tau_{a}$=0.65 GPa is the applied shear stress obtained from the ultimate tensile (true) stress at 77 K, $\sigma_{y}$=1.36 GPa is the yield strength. Taking $\mu$=75 GPa, $b$= 0.255 nm and $\nu$=0.33, $\tau_{local}$=11.5 GPa, $\tau_{local}$ is much larger than the ideal strength ($\tau_{ideal}$) of the B2 phase typically on the order of $\frac{\mu}{30}$ to $\frac{\mu}{10}$ (∼2.7 to 8.0 GPa). Owing to the limited tensile plasticity of the B2 phase,^[^^55^^]^ highly localized strain energy within the B2 MINPs cannot be fully dissipated. Consequently, microcracks readily initiate once the deformability of B2 is exhausted after a certain degree of co-deformation. Once a microcrack nucleates within a hard B2 MINP—where crack-arrest capability is intrinsically limited—it propagates rapidly across the particle and ultimately triggers premature failure.^[^^55^^]^

**Figure S1. Analysis of diffusion dynamics.** a) The difference of temperature dependent diffusivity *D* between Al and Cr solutes.^[^^56^^]^ As the fitting curves show, Cr solutes diffuse faster than Al solutes in the bcc-Fe matrix and much slower in the fcc-Fe matrix at 800 °C. b) The one-dimensional profiles of STEM-EDS image inside the structurally complex B2 MINPs of the CP sample, showing the broad diffusion interface between the shell and core.

**Figure S2**. **Formation mechanism analysis of the complex nanostructures in CP sample.** a) The formation energy per atom of the chemical ordered vs. disordered structures of the matrix, precipitates and nanocores, where a greater negative value represents a stronger atomic bonding. b) The respective ordering energy per atom and the insets show the ordering crystallographic structure and site occupancy. Note: we built three different models by changing the placement atoms in a unit cell, and we calculated the average value of formation and ordering energy. The results from the three parallel samples are consistent and negligible errors. c) The formation mechanism of the chemical heterogeneities and nanocores driven by Cr diffusion and spinodal decomposition.

**Figure S3.** **The thermo-mechanical processing and EBSD analysis.** a) The respective thermo-mechanical processing routes for the DL (Route 1), OP (Route 2) and CP (Route 3) samples. b-d) The phase distribution map, where fcc is labelled in aqua and bcc in fuchsia and the corresponding orientation relationship boundaries for the DL, OP and CP samples, respectively. e-g) Corresponding kernel average misorientation (KAM) maps for DL, OP and CP samples, respectively.

**Figure S4. The TEM chracteristics of rod B2 MINPs in the CP sample.** a) The high-mag bright-field TEM image of rod B2 MINPs. b) The EDS one-dimensional profiles across one rod B2 precipitate. c) The low-mag bright-field TEM image of rod B2 MINPs. d) The selected area electron diffraction (SAED) pattern of region SAI in (c) shows the fcc matrix. e) The SAED pattern of region SAII in (c) shows the B2 MINPs structure. f) The high-resolution TEM image of the rod B2 MINPs shows the orientation relationship and misfit between the B2 and fcc matrix, and insets are the FFT patterns. g) The SAED pattern of region SAIII in (c) shows the orientation relationship between the B2 and fcc matrix. h) The corresponding EDS mapping of (a) shows the Ni/Al rich rod B2 MINPs.

**Figure S5. The Ashby maps and the fracture toughness tests.** a) A comparison of *ε*_u_ *vs. σ*_y_ at 77 K among the present alloys with other strong and ductile alloys together with typical advanced cryogenic steels. b) A comparison of *ε*_u_ *vs. σ*_UTS_ at 77 K. See more details in Table S3. c) The *J*-integral fracture resistance curves at 298 K. The *J-R* curve was measured from single-edge bend specimens. d) A comparison of the fracture toughness *vs.* yield strength among present alloy and other strong and tough alloys at 298 K. Detailed properties of these compared materials can be found in Table S4.

**Figure S6. The analysis of *in situ* neutron diffraction of the CP sample.** a) The evolution of lattice strain against macroscopic true stress for representative fcc, bcc and B2 crystallographic plane families along both the loading direction (LD) and transverse direction (TD), respectively. b) All the in-situ neutron diffraction patterns during tension. c) The selected neutron diffraction patterns at different strains. Black crosses indicate observations, red line represents the calculated fit by Rietveld refinement.

**Figure S7.** a) High resolution HAADF-STEM image of nanocores in B2 precipitate after 4% tensile strain. b) The corresponding lattice strain distribution.

**Figure S8. The analysis of dislocations Burgers vector of B2 MINPs in bcc matrix in the fractured OP and CP samples at 77 K.** a) The visible dislocations in the ordinary B2 MINPs under ***g***=100 two-beam condition. b) The visible dislocations in the ordinary B2 MINPs under ***g***=010 two-beam condition. **Note:** The <111> dislocations will be always visible under ***g***=<100>-type two-beam condition, the change of the contrast under the two conditions exhibiting **the <001>-dominated dislocations**. c) HAADF-STEM image of the structurally complex B2 MINPs. d) The invisible <111> dislocations in the structurally complex B2 MINPs under ***g***=101 two-beam condition. **Note:** The <100> dislocations will be always visible under ***g***=<1$\bar{1}$2>-type two-beam condition (***g·b***≠0), the totally invisible (***g·b*=**0) of the dislocations in (d) exhibiting **the <111>-dominated dislocations.**

**Figure S9. The analysis of deformed substructures of fcc matrix in the fractured OP and CP samples at 298 and 77 K.** a) The dislocations tangling in the fcc matrix of the fractured OP sample at 298 K. b) The deformation twins (DTs) in the fcc matrix of the fractured OP sample at 77 K. c) The DTs in the fcc matrix of the fractured CP sample at 298 K. d) The stacking faults (SFs) and Lomer-Cottrell (L-C) lock in the fcc matrix of the fractured CP sample at 298 K. e) The more dense DTs in the fcc matrix of the fractured CP sample at 77 K. f) The selected area electron diffraction (SAED) of the DTs in the fractured CP sample at 77 K.

**Figure S10.** The strength contribution at 298 K (a) and 77 K (b) and the fracture morphology of the OP (c) and CP (d) samples at 298 K.

**Figure S11. The load-unload-reload (LUR) tests of OP and CP samples at 298/77 K.** a) The LUR curves at 298 K b) The evolution of the effective stress and the HDI stress at 77 K c) The LUR curves at 77 K, insets are the enlarged hysteresis loop images at the yield point d) The values of the effective stress and the HDI stress of the OP and CP samples at 77 K.

**Figure S12.** The microcracking behavior of B2 MINPs in CP sample at 77 K.

**Table S1.** Mixing enthalpy between Ni, Cr, Fe and Al^[^^57^^]^

| Mixing enthalpy | Fe | Ni | Cr | Al |
| --- | --- | --- | --- | --- |
| Fe | / | -2 kJ/mol | -1 kJ/mol | -11 kJ/mol |
| Ni |  | / | -7 kJ/mol | -22 kJ/mol |
| Cr |  |  | / | -10 kJ/mol |
| Al  Atomic radius | 140 pm | 135 pm | 140 pm | /  125 pm |

**Table S2.** Detailed statistical mechanical properties at 298 K in Ashby map of Fig 3c.

| Brand | Composition | σ_y_/MPa | σ_UTS_/MPa | ε_u_/% | ε_f_/% | Ref. |
| --- | --- | --- | --- | --- | --- | --- |
| 2101 | Fe-21Cr-1.5Ni-0.3Mo-5Mn-0.3Cu-0.2N | 500 | 710 |  | 43 | ^[^^58^^]^ |
| 2205 | Fe-22Cr-5Ni-3Mo-2Mn-0.2N | 950 | 1071.3 | 7 | 16 | ^[^^59^^]^ |
|  |  | 560.8 | 868.7 | 22.9 | 39.7 |  |
|  |  | 535.5 | 836.7 | 24.4 | 42.1 |  |
|  |  | 517.1 | 812.1 | 25.3 | 41.8 |  |
|  |  | 531.7 | 824.4 | 25.2 | 43.4 |  |
| 2209 | Fe-22Cr-9Ni-3Mo-1Mn-0.15N | 510 | 700 | 21 | 25 | ^[^^60^^]^ |
|  |  | 455 | 717 | 24 | 28 |  |
| 22Cr | Fe-23Cr-7Ni-3Mo-1.5Mn-0.15N | 705 | 853 | 14 | 18 | ^[^^61^^]^ |
|  |  | 722 | 854 | 14 | 17 |  |
|  |  | 481 | 751 | 19 | 23 |  |
|  |  | 486 | 753 | 18 | 22 |  |
| 2507 | Fe-25Cr-7Ni-4Mo-1Mn-0.25N | 850 | 1000 | 14 | 22 | ^[^^62^^]^ |
|  |  | 800 | 900 | 13 | 25 |  |
| 304 | Fe-18Cr-8Ni-2Mn | 258 | 579 | 70 | 75 | ^[^^63^^]^ |
|  |  | 570 | 758 | 38 | 47 |  |
|  |  | 743 | 884 | 25 | 35 |  |
| Alloy 20 | Fe-20Cr-28Ni-2.5Mo-3Cu-1.5Mn-3Cu | 294 | 578 |  | 39 | ^[^^64^^]^ |
| 904L | Fe-20Cr-23Ni-4Mo-1.3Cu-1Mn | 335 | 655 |  | 56.8 | ^[^^65^^]^ |
| S32750 | Fe-25Cr-6.5Ni-3.6Mo-0.2Cu-0.9Mn | 492 | 875 | 25 | 39.8 |  |
| ER2553 | Fe-25.5Cr-5.5Ni-3.4Mo-2Cu-1.5Mn | 382.5 | 692 | 25 | 36.2 |  |
| 6%Mo | Fe-18Cr-18Ni-6Mo-0.75Cu-0.8Mn-0.2N | 295 | 500 | 39 | 46 | ^[^^66^^]^ |
|  |  | 500 | 520 | 34 | 46 |  |
|  |  | 640 | 800 | 26 | 28 |  |
|  |  | 850 | 1000 | 7.5 | 8 |  |
|  |  | 1800 | 1900 | 1.5 | 2 |  |
| 2304 | Fe-23Cr-4.2Ni-0.3Mo-1.5Mn-0.5Cu | 600 | 753 | 45 | 57 | ^[^^67^^]^ |
|  |  | 706 | 796 | 37 | 38 |  |
|  |  | 820 | 867 | 25 | 29 |  |
|  |  | 878 | 922 | 19 | 20 |  |
|  |  | 929 | 960 | 9 | 16 |  |
| 2101 | Fe-21Cr-1.6Ni-0.2Mo-5.3Mn-0.2Cu-0.2N | 1267.3 | 1335.5 | 1.5 | 7.5 | ^[^^68^^]^ |
|  |  | 949.2 | 1098.7 | 10 | 15.6 |  |
|  |  | 622.5 | 877.8 | 40 | 52.5 |  |
| 2003 | Fe-20Cr-3.5Ni-1.9Mo-1.5Mn-0.2N | 450 | 655 |  | 25 | ^[^^69^^]^ |
| Z100 | Fe-25Cr-7Ni-3.8Mo-0.2Cu-0.8Mn-0.3N | 800 | 1150 | 23 | 26 | ^[^^70^^]^ |
|  |  | 900 | 1100 | 20 | 24 |  |
|  |  | 970 | 1020 | 13 | 16 |  |
|  | Fe-4Ni-2Si-0.4C | 940 | 1172 | 12.2 | 17.9 | ^[^^71^^]^ |
|  |  | 1111 | 1251 | 8.3 | 15.4 |  |
|  |  | 1358 | 1546 | 3.1 | 12.1 |  |
|  |  | 1471 | 1787 | 3.7 | 11.1 |  |
|  | Fe-3Mn-2Si-0.2C | 884 | 1305 | 8 | 12.1 |  |
|  |  | 982 | 1303 | 6.5 | 12 |  |
|  |  | 1122 | 1396 | 5.5 | 11.5 |  |
|  |  | 1133 | 1440 | 5 | 10.9 |  |
|  | Fe-14Co-10Ni-2Cr-1Mo-0.16C | 1398 | 1757 |  | 12 | ^[^^72^^]^ |
|  |  | 1527 | 1699 |  | 18 |  |
|  | Fe-21Cr-7Ni-2.5Mo-1.5Cu-1.65Mn-0.5Si | 920 | 1080 | 4 | 4 | ^[^^72^^]^ |
|  |  | 720 | 1010 | 13 | 13 |  |
|  |  | 420 | 720 | 27 | 35 |  |
|  |  | 410 | 720 | 25 | 32 |  |
|  |  | 460 | 670 | 23 | 30.5 |  |
|  |  | 420 | 620 | 17 | 22 |  |
|  |  | 400 | 615 | 24 | 35 |  |
|  | Fe-0.15Mn-0.03Al-0.08Ti-0.007Si-0.003C | 660 | 680 | 3 | 6 | ^[^^73^^]^ |
|  |  | 550 | 560 | 2 | 7.5 |  |
|  |  | 520 | 540 | 2 | 6 |  |
|  |  | 420 | 420 | 15 | 25 |  |
|  |  | 350 | 360 | 20 | 32.5 |  |
|  |  | 140 | 270 | 35 | 44 |  |
|  |  | 140 | 250 | 30 | 64 |  |
|  | Fe-1.5Mn-0.2Si-0.03Al-0.2C | 445 | 870 | 7.2 | 7.7 | ^[^^74^^]^ |
|  |  | 483 | 964 | 7.4 | 8.9 |  |
|  |  | 525 | 1037 | 7.1 | 7.3 |  |
|  |  | 619 | 1005 | 8.6 | 11.4 |  |
|  |  | 578 | 633 | 7.3 | 13.3 |  |
|  | Fe-1Mn-0.25Si-0.1V-0.15C | 510 | 843 | 9.8 | 13.5 | ^[^^75^^]^ |
|  |  | 581 | 978 | 9.3 | 17.6 |  |
|  |  | 540 | 1044 | 11.5 | 18.1 |  |
|  |  | 565 | 1015 | 10.4 | 16.6 |  |
|  | Fe-0.83Mn-0.3V-0.22Si-0.1C | 650 | 830 | 9 | 18 | ^[^^76^^]^ |
|  |  | 580 | 720 | 10 | 20 |  |
|  |  | 480 | 650 | 11 | 22 |  |
|  |  | 630 | 790 | 10 | 22 |  |
|  |  | 720 | 850 | 10 | 20 |  |
|  |  | 700 | 810 | 9 | 20 |  |
|  | Fe-13Cr-4.5Al | 370 | 440 | 15 | 32.5 | ^[^^77^^]^ |
|  |  | 676 | 770 | 5 | 14 |  |
|  | Fe-13Cr-4.5Al-2Mo-1Nb | 440 | 540 | 15 | 31.5 |  |
|  |  | 928 | 1066 | 5.8 | 11.2 |  |
|  | Fe-13Cr-4.5Al-2Mo-1Nb-0.2Si | 490 | 600 | 13 | 25 |  |
|  |  | 1012 | 1197 | 7 | 12.8 |  |
| Q&P | Fe-2.5Mn-0.8Cr-1.5Si-0.3C | 1211 | 1448 | 6.5 | 12.6 | ^[^^31^^]^ |
|  |  | 1227 | 1437 | 6.1 | 13.2 |  |
|  |  | 1441 | 1631 | 3.6 | 8.1 |  |
| MMS | Fe-9.3Mn-2Al-0.2C | 750 | 1400 | 25 | 25 | ^[^^78^^]^ |
|  |  | 1000 | 1300 | 10 | 20 |  |
| 18Ni | Fe-18.4Ni-0.21Cr-3.2Mo-1.55Ti-0.07Si | 1873 | 1939 | 2 | 6.4 | ^[^^79^^]^ |
|  |  | 1751 | 1838 | 2 | 6.5 |  |
|  |  | 1422 | 1567 | 6 | 11.1 |  |
|  |  | 787 | 1358 | 9.5 | 15.7 |  |
| FeMnAlC | Fe-21Mn-6Al-1C | 289 | 641 | 46 | 63 | ^[^^80^^]^ |
| TRIP/TWIP | Fe-21Mn-6Al-1.7Si-1C | 343 | 720 | 64 | 80 |  |
|  |  | 442 | 680 | 30 | 30 |  |
|  | Fe-21Mn-6Al-3.3Si-1C | 436 | 836 | 78 | 95 |  |
|  |  | 1200 | 1250 | 3 | 10 |  |
|  | Fe-30Mn-9Al-1.2C | 450 | 750 | 70 | 80 | ^[^^81^^]^ |
|  | Fe-30Mn-9Al-0.7Si-1.2C | 700 | 800 | 46 | 53 |  |
|  | Fe-30Mn-9Al-1.6Si-1.2C | 950 | 1000 | 15 | 22 |  |

**Table S3.** Detailed statistical mechanical properties at 77 K in Ashby map of Fig. 3d.

| Alloys | σ_y_/MPa | σ_UTS_/MPa | ε_u_/% | ε_f_/% | Ref. |
| --- | --- | --- | --- | --- | --- |
| Fe-based MEAs | 496  1096  914  495  546  723  798 | 1594  1680  1517  993  1960  1380  1595 | 45  52  53  62  31  60  40 | 46  53  54  62  31  60  40 | ^[^^82^^]^ |
| TWIP steels | 760 | 1312 | 56 | 56 | ^[^^83^^]^ |
| TRIP steels | 680  646  759  811 | 1477  1509  1355  1403 | 25.4  53.5  40.7  66.2 | 25.4  53.5  40.7  67.2 | ^[^^84^^]^ |
| M/HEAs | 440  495  930  672  783  450  511  965  995  657 | 993  1097  1555  1277  1359  1026  1004  1397  1577  1228 | 61  65.5  51.7  74.5  80.5  81.5  67.4  21.6  39.8  68.6 | 61  66  52  75  81  82  68  22  40  69 | ^[^^82^^]^ |
| 304L SS | 420 | 1984 | 26 | 40 | ^[^^85^^]^ |
| 316LN SS | 793 | 1314 | 55 | 58 | ^[^^86^^]^ |
| High Ni steels | 954  904  1027  1086 | 1083  1093  1036  1094 | 17  20  10  6 | 36.2  36.9  30.3  33.3 | ^[^^87^^]^ |
| Duplex SS | 1050 | 1150 | 30 | 43 | ^[^^88^^]^ |
|  | 1615 | 1650 | 29 | 30 | ^[^^89^^]^ |
| FCC+B2 | 790  810  747  690 | 1320  1306  936  1045 | 30  26  66  8 | 31  26  66  8 | ^[^^55^^]^ |
| FCC+L12 | 1078  863  1020 | 1690  1580  1455 | 51  45  26 | 51  45  26 | ^[^^55^^]^ |
| Lüders TRIP duplex steel | 1640 | 2109 | 29.1 | 31 | ^[^^82^^]^ |

**Table S4.** Detailed statistical mechanical properties of Fig. S5d.

| Alloy | Phases | σ_y_/MPa | *J_Ic_*/kJ/m^2^ | K*_JIc_*/MPa·m^0.5^ | Ref. |
| --- | --- | --- | --- | --- | --- |
| 2564 | α, γ | 647 | 200 |  | ^[^^90^^]^ |
|  |  | 631 | 200 |  |  |
| HCHS | α, γ, Carbides | 1800 |  | 20 | ^[^^25^^]^ |
|  |  | 1550 |  | 37 |  |
|  |  | 988 |  | 40 |  |
| TRIP-assisted MPS | α, γ, α_b_ | 600 | 130 |  | ^[^^33^^]^ |
|  |  | 600 | 220 |  |  |
|  |  | 700 | 60 |  |  |
|  |  | 850 | 90 |  |  |
|  |  | 900 | 40 |  |  |
|  |  | 1100 | 90 |  |  |
| Medium Mn steels |  | 1978 | 46.9 | 101.2 | ^[^^24^^]^ |
|  |  | 1714 | 19.6 | 65.4 |  |
|  |  | 750 |  | 73.9 | ^[^^78^^]^ |
|  |  | 1650 |  | 87.5 |  |
|  |  | 1000 |  | 89.9 |  |
| TRIP steel |  | 1177 |  | 86 | ^[^^32^^]^ |
|  |  | 1121 |  | 129 |  |
|  |  | 1125 |  | 141 |  |
|  |  | 1147 |  | 154 |  |
|  |  | 857 |  | 71 |  |
|  |  | 805 |  | 54 |  |
| Q&P steels |  | 1145 |  | 100 | ^[^^31^^]^ |
|  |  | 1211 |  | 60 |  |
|  |  | 1441 |  | 70 |  |
| Bainite | BCC | 1400 |  | 30 | ^[^^26^^]^ |
|  |  | 1480 |  | 38 |  |
|  |  | 1200 |  | 42 |  |
| DP steels |  | 560 |  | 41 | ^[^^34^^]^ |
|  |  | 615 |  | 58 |  |
|  |  | 660 |  | 67 |  |
|  |  | 710 |  | 79 |  |
| Martensitic SS | BCC | 1147 |  | 62 | ^[^^29^^]^ |
|  |  | 1182 |  | 69 |  |
| AISI 4340 | BCC | 1500 |  | 90 | ^[^^28^^]^ |
|  |  | 1600 |  | 70 |  |
|  |  | 1500 |  | 80 |  |
|  |  | 1300 |  | 120 |  |
|  |  | 1200 |  | 160 |  |
|  |  | 900 |  | 170 |  |
| 300-M Martensite steels | BCC | 1497 |  | 35.5 | ^[^^27^^]^ |
|  |  | 1737 |  | 65.1 |  |
|  |  | 1074 |  | 152 |  |
| 18Ni Maraging steels | BCC | 1939 |  | 76 | ^[^^30^^]^ |
|  |  | 1838 |  | 97 |  |
|  |  | 1567 |  | 166 |  |
| EHEA | γ, B2 | 730 | 400 | 306 | ^[^^36^^]^ |
| CrCoNi | γ | 440 | 211.6 | 207.7 | ^[^^37^^]^ |
| FeCrMnCoNi | γ | 400 | 250 | 217 | ^[^^35^^]^ |

**Table S5.** The strength contributions in the fcc/bcc phase of CP/OP sample at 298 K.

| Phase | Solid solution | Grain boundary | Dislocation | Precipitation | All | ROM |
| --- | --- | --- | --- | --- | --- | --- |
| CP-fcc | 130 MPa | 133 MPa | 303 MPa | 71 MPa | 637 MPa | 750 |
| CP-bcc | 325 MPa | 235 MPa | 265 MPa | 93.5 MPa | 918.5 MPa | MPa |
| OP-fcc | 130 MPa | 118 MPa | 287 MPa | 43.8 MPa | 578.8 MPa | 701 |
| OP-bcc | 325 MPa | 239 MPa | 271 MPa | 50.5 MPa | 885.5 MPa | MPa |

**Table S6.** The strength contributions in the fcc/bcc phase of CP/OP sample at 77 K.

| Phase | Solid solution | Grain boundary | Dislocation | Precipitation | All | ROM |
| --- | --- | --- | --- | --- | --- | --- |
| CP-fcc | 213 MPa | 133 MPa | 323 MPa | 71 MPa | 740 MPa | 900 |
| CP-bcc | 529 MPa | 235 MPa | 282 MPa | 93.5 MPa | 1139.5 MPa | MPa |
| OP-fcc | 213 MPa | 118 MPa | 306 MPa | 43.8 MPa | 680.8 MPa | 851 |
| OP-bcc | 529 MPa | 239 MPa | 289 MPa | 50.5 MPa | 1107.5 MPa | MPa |

**Table S7.** Bulk chemical composition of the as-cast alloy by the ICP-OES method.

| Elements | Fe | Ni | Cr | Al |
| --- | --- | --- | --- | --- |
| wt.% | Bal. | 17.70 | 15.74 | 4.94 |
| at.% | Bal. | 15.95 | 16.01 | 9.68 |

**Supporting Information References**

[1]	S. He, O. I. Gorbatov, P. Peng, *Calphad* **2023**, 82, 102591.

[2]	G. Kresse, J. Furthmüller, *Comput. Mater. Sci.* **1996**, 6, 15.

[3]	G. Kresse, J. Hafner, *Phys. Rev. B* **1993**, 47, 558.

[4]	J. P. Perdew, K. Burke, M. Ernzerhof, *Phys. Rev. Lett.* **1996**, 77, 3865.

[5]	M. G. Tucker, D. A. Keen, M. T. Dove, A. L. Goodwin, Q. Hui, *J. Phys.: Condens. Matter* **2007**, 19, 335218.

[6]	G. K. Williamson, W. H. Hall, *Acta Metall.* **1953**, 1, 22.

[7]	R. Dakhlaoui, C. Braham, A. Baczmański, *Mater. Sci. Eng. A* **2007**, 444, 6.

[8]	T. Ungar, I. Dragomir, A. Revesz, A. Borbely, *J. Appl. Crystallogr.* **1999**, 32, 992.

[9]	Y. Shi, S. Li, T. L. Lee, X. Hui, Z. Zhang, R. Li, M. Zhang, S. Kabra, Y.-D. Wang, *Mater. Sci. Eng. A* **2020**, 771, 138555.

[10]	L. Ma, L. Wang, Z. Nie, F. Wang, Y. Xue, J. Zhou, T. Cao, Y. Wang, Y. Ren, *Acta Mater.* **2017**, 128, 12.

[11]	T. Maki, T. Furuhara, K. Tsuzaki, *ISIJ Int.* **2001**, 41, 571.

[12]	R. Feng, Y. Rao, C. Liu, X. Xie, D. Yu, Y. Chen, M. Ghazisaeidi, T. Ungar, H. Wang, K. An, P. K. Liaw, *Nat. Commun.* **2021**, 12, 3588.

[13]	J. C. Rao, H. Y. Diao, V. Ocelík, D. Vainchtein, C. Zhang, C. Kuo, Z. Tang, W. Guo, J. D. Poplawsky, Y. Zhou, P. K. Liaw, J. T. M. De Hosson, *Acta Mater.* **2017**, 131, 206.

[14]	E. Clouet, L. Laé, T. Épicier, W. Lefebvre, M. Nastar, A. Deschamps, *Nat. Mater.* **2006**, 5, 482.

[15]	A. Orthacker, G. Haberfehlner, J. Taendl, M. C. Poletti, B. Sonderegger, G. Kothleitner, *Nat. Mater.* **2018**, 17, 1101.

[16]	S. Jiang, Y. Xu, R. Wang, X. Chen, C. Guan, Y. Peng, F. Liu, M. Wang, X. Liu, S. Zhang, G. Tian, S. Jin, H. Wang, H. Toda, X. Jin, G. Liu, B. Gault, J. Sun, *Nature* **2025**, 641, 358.

[17]	Y. Kawahara, A. Tokuhisa, T. Maeda, H. Shirahata, R. Uemori, K. Kaneko, *Scr. Mater.* **2024**, 249, 116169.

[18]	J. B. Seol, S. H. Na, B. Gault, J. E. Kim, J. C. Han, C. G. Park, D. Raabe, *Sci. Rep.* **2017**, 7, 42547.

[19]	P. A. Korzhavyi, A. V. Ruban, A. Y. Lozovoi, Y. K. Vekilov, I. A. Abrikosov, B. Johansson, *Phys. Rev. B* **2000**, 61, 6003.

[20]	A. J. Ardell, *Acta Metall.* **1972**, 20, 61.

[21]	J. Charkhchian, A. Zarei-Hanzaki, A. Moshiri, H. R. Abedi, T. M. Schwarz, R. Lawitzki, G. Schmitz, K. Chadha, C. Aranas Jr, J. Shen, J. P. Oliveira, *Adv. Eng. Mater.* **2023**, 25, 2300164.

[22]	Z. Geng, C. Chen, M. Song, J. Luo, J. Chen, R. Li, K. Zhou, *J. Mater. Sci. Technol.* **2024**, 187, 141.

[23]	A. Standard, ASTM E1820-17a 2018.

[24]	L. Liu, Q. Yu, Z. Wang, J. Ell, M. X. Huang, O. Ritchie Robert, *Science* **2020**, 368, 1347.

[25]	S. K. Putatunda, *Mater. Sci. Eng. A* **2001**, 297, 31.

[26]	C. Garcia-Mateo, F. G. Caballero, *ISIJ Int.* **2005**, 45, 1736.

[27]	R. O. Ritchie, *J Eng Mater Tech* **1977**, 99, 195.

[28]	M. Manokaran, A. S. Kashinath, J. S. Jha, S. P. Toppo, R. P. Singh, *J. Mater. Eng. Perform.* **2020**, 29, 6748.

[29]	D. M. Jafarlou, C. Walde, V. K. Champagne, S. Krishnamurty, I. R. Grosse, *Mater. Des.* **2018**, 155, 134.

[30]	B. Wang, Q. Q. Duan, P. Zhang, Z. J. Zhang, X. W. Li, Z. F. Zhang, *Mater. Sci. Eng. A* **2020**, 771, 138553.

[31]	Z. Xiong, P. J. Jacques, A. Perlade, T. Pardoen, *Metall. Mater. Trans. A* **2019**, 50, 3502.

[32]	J. Kobayashi, D. Ina, A. Futamura, K.-i. Sugimoto, *ISIJ Int.* **2014**, 54, 955.

[33]	G. Lacroix, T. Pardoen, P. J. Jacques, *Acta Mater.* **2008**, 56, 3900.

[34]	M. E. Haque, K. V. Sudhakar, *Int. J. Fatigue* **2002**, 24, 1003.

[35]	B. Gludovatz, A. Hohenwarter, D. Catoor, E. H. Chang, E. P. George, R. O. Ritchie, *Science* **2014**, 345, 1153.

[36]	P. Kumar, S. Huang, D. H. Cook, K. Chen, U. Ramamurty, X. Tan, R. O. Ritchie, *Nat. Commun.* **2024**, 15, 841.

[37]	B. Gludovatz, A. Hohenwarter, K. V. S. Thurston, H. Bei, Z. Wu, E. P. George, R. O. Ritchie, *Nat. Commun.* **2016**, 7, 10602.

[38]	C. Varvenne, A. Luque, W. A. Curtin, *Acta Mater.* **2016**, 118, 164.

[39]	B. Yin, F. Maresca, W. Curtin, *Acta Mater.* **2020**, 188, 486.

[40]	D. Zhang, H. Wang, J. Zhang, H. Xue, G. Liu, J. Sun, *J. Mater. Sci. Technol.* **2021**, 87, 184.

[41]	P. Pandey, S. Kashyap, D. Palanisamy, A. Sharma, K. Chattopadhyay, *Acta Mater.* **2019**, 177, 82.

[42]	F. Maresca, W. A. Curtin, *Acta Mater.* **2020**, 182, 235.

[43]	Y. Zhang, C. Wang, K. M. Reddy, W. Li, X. Wang, *Acta Mater.* **2022**, 226, 117670.

[44]	S. H. Gao, J. Y. Zhang, H. Wang, S. Y. Liu, J. Kuang, J. Li, G. Liu, J. Sun, *Scr. Mater.* **2025**, 267, 116837.

[45]	B. Yin, F. Maresca, W. A. Curtin, *Acta Mater.* **2020**, 188, 486.

[46]	M.-Y. Seok, I.-C. Choi, J. Moon, S. Kim, U. Ramamurty, J.-i. Jang, *Scr. Mater.* **2014**, 87, 49.

[47]	Y. Wang, J. Sun, T. Jiang, Y. Sun, S. Guo, Y. Liu, *Acta Mater.* **2018**, 158, 247.

[48]	D. Jorge-Badiola, A. Iza-Mendia, I. Gutiérrez, *Mater. Sci. Eng. A* **2005**, 394, 445.

[49]	C. Zhu, T. Harrington, G. T. Gray, K. S. Vecchio, *Acta Mater.* **2018**, 155, 104.

[50]	G. Liu, G. J. Zhang, F. Jiang, X. D. Ding, Y. J. Sun, J. Sun, E. Ma, *Nat. Mater.* **2013**, 12, 344.

[51]	F. Wang, M. Song, M. N. Elkot, N. Yao, B. Sun, M. Song, Z. Wang, D. Raabe, *Science* **2024**, 384, 1017.

[52]	G. Sun, M. Lei, S. Liu, B. Wen, *Comput. Mater. Sci.* **2024**, 233, 112720.

[53]	P. M. Anderson, J. P. Hirth, J. Lothe, *Theory of dislocations*, Cambridge University Press, **2017**.

[54]	C. X. Huang, Y. F. Wang, X. L. Ma, S. Yin, H. W. Höppel, M. Göken, X. L. Wu, H. J. Gao, Y. T. Zhu, *Mater. Today* **2018**, 21, 713.

[55]	D. D. Zhang, J. Y. Zhang, J. Kuang, G. Liu, J. Sun, *Acta Mater.* **2022**, 233, 117981.

[56]	Z. Balogh, G. Schmitz, in *Physical metallurgy*, Elsevier, 2014.

[57]	A. Takeuchi, A. Inoue, *Mater. Trans.* **2005**, 46, 2817.

[58]	L. Ma, S. Hu, J. Shen, *J. Mater. Eng. Perform.* **2017**, 26, 250.

[59]	S. Papula, M. Song, A. Pateras, X.-B. Chen, M. Brandt, M. Easton, Y. Yagodzinskyy, I. Virkkunen, H. Hänninen, *Materials*, 10.3390/ma12152468

[60]	G. Posch, K. Chladil, H. Chladil, *Weld. World* **2017**, 61, 873.

[61]	A. Baghdadchi, V. A. Hosseini, M. A. Valiente Bermejo, B. Axelsson, E. Harati, M. Högström, L. Karlsson, *J. Mater. Sci.* **2022**, 57, 9556.

[62]	C. Y. Ma, L. Zhou, R. X. Zhang, D. G. Li, F. Y. Shu, X. G. Song, Y. Q. Zhao, *J. Mater. Res. Technol.* **2020**, 9, 8296.

[63]	M. Milad, N. Zreiba, F. Elhalouani, C. Baradai, *J. Mater. Process. Tech.* **2008**, 203, 80.

[64]	S. Idapalapati, K. M. Kelvin Loh, S. Yeo, *Eng. Fail. Anal.* **2017**, 78, 99.

[65]	K. D. Ramkumar, A. Dagur, A. Kartha, M. Subodh, V. Cheekati, A. Duraisamy, M. G. Kumar, W. Sunny, A. Chatterjee, J. Abraham, J. Abraham, *J. Manuf. Process.* **2017**, 30.

[66]	T.-T. Chen, J. Wang, Y. Zhang, P. Jiang, F.-P. Yuan, P.-D. Han, X.-L. Wu, *Mater. Sci. Eng. A* **2022**, 837, 142727.

[67]	D. G. Rodrigues, G. G. B. Maria, N. A. L. Viana, D. B. Santos, *Mater. Charact.* **2019**, 150, 138.

[68]	X. Zhang, J. Li, W. Mu, J. Gu, S. Dai, Z. Zhao, *Mater. Sci. Eng. A* **2022**, 838, 142731.

[69]	R. Francis, G. Byrne, *Metals* **2021**, 11, 836.

[70]	C. Gennari, L. Pezzato, G. Tarabotti, A. Zambon, A. Di Schino, I. Calliari, *Materials*, 10.3390/ma13071613

[71]	V. T. T. Miihkinen, D. V. Edmonds, *Mater. Sci. Technol.* **1987**, 3, 432.

[72]	K. Unnikrishnan, A. K. Mallik, *Mater. Sci. Eng.* **1987**, 94, 175.

[73]	S. S. Hazra, E. V. Pereloma, A. A. Gazder, *Acta Mater.* **2011**, 59, 4015.

[74]	M. Bechtold, Y. Adachi, D. Ponge, D. Raabe, *Acta Mater.* **2010**, 59, 658.

[75]	Y. I. Son, Y. K. Lee, K.-T. Park, C. S. Lee, D. H. Shin, *Acta Mater.* **2005**, 53, 3125.

[76]	N. Kamikawa, K. Sato, G. Miyamoto, M. Murayama, N. Sekido, K. Tsuzaki, T. Furuhara, *Acta Mater.* **2015**, 83, 383.

[77]	S. Y. Liu, J. Y. Zhang, H. Zhang, H. Xue, H. Wang, G. Liu, J. Sun, *Int. J. Plast.* **2022**, 103438.

[78]	C. Hu, C. P. Huang, Y. X. Liu, A. Perlade, K. Y. Zhu, M. X. Huang, *Acta Mater.* **2023**, 245, 118629.

[79]	B. Wang, P. Zhang, Q. Q. Duan, Z. J. Zhang, H. J. Yang, X. W. Li, Z. F. Zhang, *Mater. Sci. Eng. A* **2017**, 707, 674.

[80]	H. Zhi, J. Li, W. Li, M. Elkot, S. Antonov, H. Zhang, M. Lai, *Acta Mater.* **2023**, 245, 118611.

[81]	Z. Wang, W. Lu, H. Zhao, J. He, K. Wang, B. Zhou, D. Ponge, D. Raabe, Z. Li, *Acta Mater.* **2020**, 198, 258.

[82]	Q. Gao, R. Wei, S. Feng, C. Chen, Z. Han, L. Chen, T. Wang, S. Wu, F. Li, *Scr. Mater.* **2023**, 228, 115334.

[83]	L. Tang, L. Wang, M. Wang, H. Liu, S. Kabra, Y. Chiu, B. Cai, *Acta Mater.* **2020**, 200, 943.

[84]	S. S. Sohn, S. Hong, J. Lee, B.-C. Suh, S.-K. Kim, B.-J. Lee, N. J. Kim, S. Lee, *Acta Mater.* **2015**, 100, 39.

[85]	C. Zheng, W. Yu, *Mater. Sci. Eng. A* **2018**, 710, 359.

[86]	P. Czarkowski, A. T. Krawczynska, R. Slesinski, T. Brynk, J. Budniak, M. Lewandowska, K. J. Kurzydlowski, *Fusion Eng. Des.* **2011**, 86, 2517.

[87]	H.-S. Shin, H.-M. Lee, M.-S. Kim, *Int. J. Impact Eng.* **2000**, 24, 571.

[88]	N. Koga, T. Nameki, O. Umezawa, V. Tschan, K.-P. Weiss, *Mater. Sci. Eng. A* **2021**, 801, 140442.

[89]	M. Chen, J. He, J. Li, H. Liu, S. Xing, G. Wang, *Mater. Sci. Eng. A* **2022**, 831, 142335.

[90]	O. Kolednik, M. Albrecht, M. Berchthaler, H. Germ, R. Pippan, F. Riemelmoser, J. Stampfl, J. Wei, *Acta Mater.* **1996**, 44, 3307.
